# Ranolazine Inhibits Pyroptosis *via* Regulation of miR-135b in the Treatment of Diabetic Cardiac Fibrosis

**DOI:** 10.3389/fmolb.2022.806966

**Published:** 2022-01-26

**Authors:** Long Ren, Xi Chen, Binyang Nie, Huan Qu, Jiaming Ju, Yunlong Bai

**Affiliations:** ^1^ Department of Pharmacology (State-Province Key Laboratories of Biomedicine- Pharmaceutics of China, Key Laboratory of Cardiovascular Research, Ministry of Education), College of Pharmacy, Harbin Medical University, Harbin, China; ^2^ Department of Pharmacy, The First Affiliated Hospital of Harbin Medical University, Harbin, China; ^3^ Bachelor of Commerce, Pharmacology and Finance Student, University of Sydney, Sydney, NSW, Australia; ^4^ Translational Medicine Research and Cooperation Center of Northern China, Heilongjiang Academy of Medical Sciences, Harbin, China; ^5^ Joint International Research Laboratory of Cardiovascular Medicine, Ministry of Education, College of Pharmacy, Harbin Medical University, Harbin, China

**Keywords:** diabetic cardiomyopathy, ranolazine, MicroRNA-135b, cardiac fibrosis, pyroptosis

## Abstract

Diabetic cardiomyopathy (DCM) is a major cardiovascular complication of diabetes mellitus (DM), and cardiac fibrosis is a characteristic pathological manifestation of DCM. DCM can be exacerbated by pyroptosis, and pyroptosis is a potential target of microRNAs (miRNAs). miR-135b is involved in delaying the progression of numerous cardiovascular diseases, Nonetheless, the role of miR-135b in diabetic cardiac fibrosis is unclear. Ranolazine is a piperazine derivative and is effective for the treatment of cardiovascular disease. The purpose of the study was to elucidate the mechanism of action of ranolazine against diabetic cardiac fibrosis and to investigate the role of miR-135b in this process. Functional and structural changes in the rat heart were examined by echocardiography, hematoxylin-eosin (H&E) and Masson staining. Immunohistochemistry was used to assess the expression of caspase-1, interleukin-1β (IL-1β), gasdermin D (GSDMD), transforming growth factor-β1 (TGF-β1), collagen I and collagen III in the rat left ventricle. Western blot and immunofluorescence were used to detect the protein expression of caspase-1, IL-1β, GSDMD, TGF-β1, collagen I and collagen III proteins, and the mRNA levels were determined using fluorescent quantitative PCR. Ranolazine reduced pyroptosis and inhibited collagen deposition, improving cardiac function in rats. Ranolazine increased miR-135b expression in high glucose-treated cardiac fibroblasts, and miR-135b directly bound to caspase-1. Interference with miR-135b reduced the effects of ranolazine on pyroptosis and collagen deposition. Ranolazine treatment of diabetic cardiac fibrosis inhibited pyroptosis and collagen deposition by upregulating miR-135b. Our study provides a solid theoretical basis for understanding the pathogenesis of diabetic cardiac fibrosis and the clinical use of ranolazine in the treatment of DCM.

## Introduction

The number of patients with diabetes mellitus (DM) has been increasing at an alarming rate for decades (Guariguata et al., 2014; Wang H et al., 2019). The latest statistics of the International Diabetes Federation indicate that approximately 451 million adults worldwide have DM, and this number is expected to reach 693 million in 2045 (Cho et al., 2018). Diabetic cardiomyopathy (DCM) is a major cardiovascular complication of diabetes that can lead to structural and functional damage of the heart independent of the high blood pressure, coronary artery disease or atherosclerosis associated with DM (de Simone et al., 2010; Holscher et al., 2016). Cardiac fibrosis is the main pathological feature of DCM and can increase the risk of heart failure, arrhythmia and sudden death (Asbun and Villarreal, 2006). Cardiac fibrosis is often accompanied by increase in the levels of inflammatory factors (Palomer et al., 2020). Pyroptosis is a form of programmed cell death associated with inflammation (Bergsbaken et al., 2009). The classical pathway of pyroptosis is mediated by caspase-1 and can increase mature interleukin-1β (IL-1β) levels (Schroder et al., 2010). Recent studies have revealed that gasdermin D (GSDMD) plays an important indicator role in pyroptosis, and the N-terminus of GSDMD creates small pores in the cell membrane, causing the release of contents and the massive entry of external substances such as water molecules, eventually lead to cell death (Kovacs and Miao, 2017). Activation of inflammatory factors and the release of cytokines, which promotes the formation of collagen deposits and fibrosis, exacerbate DCM (Bracey et al., 2014). Therefore, suppression of pyroptosis is essential for the prevention and treatment of diabetic cardiac fibrosis.

Recent studies have demonstrated a close relationship between microRNAs (miRNAs) and pyroptosis. For example, miR-138-5 inhibition induces the upregulation of Sirt1 expression to inhibit cardiomyocyte pyroptosis and delay the progression of myocardial infarction (Mao et al., 2019), and miR-30d promotes the production of myocardial pyroptosis leading to DCM (Li et al., 2014). Upregulation of miR-214 inhibits pyroptosis in the treatment of DCM (Yang et al., 2019). These observations suggest that miRNAs can influence the development and progression of cardiomyopathy by regulating pyroptosis. Overexpression of miR-135b can treat pathological myocardial hypertrophy and myocardial ischemia(Chu et al., 2018; Li et al., 2020). However, whether miR-135b affects the onset and development of DCM by regulating pyroptosis has not yet been determined.

Ranolazine is a piperazine derivative that is approved by the US Food and Drug Administration in 2006 for the treatment of stable angina pectoris. Additionally, ranolazine is effective for the treatment of other cardiovascular conditions, such as atrial fibrillation, arrhythmias and diastolic dysfunction (Banerjee et al., 2017). Previous studies have shown that ranolazine reduces the cardiotoxicity of anthraquinone anticancer drugs and decreases cardiac fibrosis (Cappetta et al., 2017). Moreover, ranolazine has been shown to promote AKT phosphorylation to inhibit cardiomyocyte inflammation and thereby improve cardiac function in a rat model of heart failure (Wang G T et al., 2019). However, the mechanism of ranolazine in diabetic cardiac fibrosis has not been fully elucidated.

The purpose of the present study was to elucidate the mechanism of ranolazine in the treatment of diabetic cardiac fibrosis and to investigate the role of miR-135b in this process. We hypothesized that ranolazine inhibits pyroptosis and collagen deposition by up-regulating miR-135b to treat DCM. Our study provides a solid theoretical basis for understanding the pathogenesis of diabetic cardiac fibrosis and the clinical use of ranolazine in the treatment of DCM.

## Materials and Methods

### Establishment of Animal Models

Sprague-Dawley (SD) rats (6–8 weeks of age, weighing 180–200 g) were purchased from the Animal Experimentation Centre of the Second Hospital of Harbin Medical University. The control group was given a standard diet (STD), and the high-fat group was given a high-fat diet [ordinary mixed feed (88.5%), cholesterol (1%), lard (10%) and pig bile salt (0.5%)]. After 8 weeks of high-fat feeding, the rats were fasted for 12–16 h and then injected intraperitoneally with 35 mg/kg streptozotocin (STZ, Sigma, United States). The control group was injected with the same amount of buffered solution, and the blood glucose levels of rats were measured 7 days later using a Roche blood glucose monitor (Roche, Germany). A fasting blood glucose level greater than 16.7 mmol/L in the high-fat diet group indicated that the DM model was successful. Ranolazine was administered at a dose of 30 mg/kg by gavage to the DM + Ranolazine (DM + Ran) group, and equal amounts of drug solvent were administered to the other groups. All animals were kept for 12 weeks under DM modeling (high-fat modeling for 20 weeks) for sampling. The rats were freely exposed to standard nutritious food and fresh sterile water, and they were maintained in an environment with a controlled temperature (22 ± 1°C) and a light-dark cycle (12 h light/12 h dark). All experimental animals were male, and the experiments were performed with the approval of the Ethics Committee of Harbin Medical University (No. IRB3005619) and maintained following the guidelines of the China Council on Animal Management for the care and use of animals.

### Echocardiographic Function

All experimental rats were measured for echocardiographic function using the using a Vevo 1100 high-resolution imaging system (Visual Sonics, Toronto, Canada) and an ultrasound instrument with a 10 MHz phased-array transducer (Vivid 7, GE Medical, Milwaukee, Wisconsin) at 12 weeks after the diabetes model was established. Rats were first anesthetized with 2% pentobarbital sodium, order to anesthetize without offending respiration. Cardiac function was evaluated by computer calculation of left ventricular ejection fraction (LVEF) and left ventricular fractional shortening (LVFS) percentages.

### Biochemical Index

After 12 h of fasting, blood was collected from the rats *via* the tail vein, and the blood was placed onto the blood glucose reagent paper, which was inserted into a Roche blood glucose meter to obtain the fasting blood glucose value. Fasting blood glucose was measured in rats every four weeks before the experimental animals were sampled. Total cholesterol (T-CHO), triglycerides (TG), low-density lipoprotein (LDL-C) and high-density lipoprotein (HDL-C) were determined by collecting blood samples *via* the tail vein using a kit (Nanjing Jiancheng Institute of Biological Engineering, China) according to the manufacturer’s instructions.

### Hematoxylin-Eosin (H&E) and Masson Staining

After sampling, the heart tissue was fixed in 4% paraformaldehyde for 48 h and placed under tap water for 3 h before dehydration. The tissue was then embedded in paraffin and then sectioned into 5 μm thick continuous sections. Staining was then performed according to the instructions of the H&E and Masson kits (Solarbio, China). Tissue sections were sealed using neutral gum, and image acquisition was performed using fluorescence microscopy (Nikon 80i, Japan) under white light conditions.

### Immunohistochemical Staining

Tissue sections were removed oxidase and then subjected to antigen repair. The sections were blocked for 30 min at room temperature using 5% bovine serum albumin followed by incubation with the following primary antibodies at 4°C overnight: caspase-1 (1:200) (Cell Signaling Technology,MA, United States), IL-1β (1:100) (Cell Signaling Technology,MA, United States), TGF-β1 (1:200) (Cell Signaling Technology, MA, United States), GSDMD (1:100) (Bioss, Beijing, China), collagen I (1:200) (Cell Signaling Technology, MA, United States) and collagen III (1:200) (Cell Signaling Technology, MA, United States). Next day, the samples were then washed three times for 5 min each using PBST, and the corresponding secondary antibodies were added followed by incubation for 1 h at room temperature. The sections were then stained with diaminobenzidine, and the nuclei were stained with hematoxylin. The sections were then sealed using neutral gum. A fluorescence microscope under white light conditions (Nikon 80i, Japan) was used for imaging.

### Immunofluorescence

Cardiac fibroblasts were fixed in 4% paraformaldehyde for 15 min followed by permeabilized with 0.1% Triton X-100 for 5 min. The samples were then blocked with 5% bovine serum albumin at room temperature for 1 h. The samples were then incubated overnight at 4°C with the following primary antibodies:caspase-1 (1:200), IL-1β (1:100), TGF-β1 (1:200), collagen I (1:200) and collagen III (1:200). And, the samples were incubated with secondary antibodies at 37°C for 1 h followed by DAPI staining for 15 min. Image acquisition was performed using a fluorescence microscope (Nikon 80i, Japan) showing the nucleus in blue, and the cytoplasm in green or red.

### Primary Cell Culture

Unweaned 1- to 3-day-old SD rats were purchased from the Animal Experiment Centre of the Second Hospital of Harbin Medical University, and cardiac fibroblasts were isolated by trypsin digestion. Cells were incubated in 5.5 mM glucose (NG group), 30 mM glucose (HG group) or 30 mM glucose with 20 μM ranolazine (HG + Ran) at 37°C and 5% CO_2_ under humid air conditions for 48 h. Cells were then transfected with anti-miR-135b oligonucleotide (AMO-135b) or the corresponding negative control (AMO-NC) designed and synthesized by RIOBIO (Guangzhou, China).

### qRT-PCR

Total RNA was extracted from tissues and cells using TRIzol (Invitrogen). cDNA was synthesized by reverse transcription according to the instructions given by the manufacturer of the reverse transcription reagent. cDNA was synthesized by real-time quantitative PCR using SYBR Green I (Yoyobo, Japan) in an ABI 7500 (Applied Biosystems, United States) rapid system, using U6 as an internal reference for miR-135b, and others used GAPDH as an internal reference. The primer sequences were as follows:

**Table:** 

Primer and interfering	RNA Sequence
miR-135b-F	GGG​GTA​TGG​CTT​TTC​ATT​CC
miR-135b-R	CAGTGCGTGTCGTGGAGT
Caspase-1-F	ATG​CCG​TGG​AGA​GAA​ACA​AG
Caspase-1-R	CCA​GGA​CAC​ATT​ATC​TGG​TG
IL-1β-F	CCT​TGT​GCA​AGT​GTC​TGA​AG
IL-1β-R	GGG​CTT​GGA​AGC​AAT​CCT​TA
TGF-β1-F	ACT​ACT​ACG​CCA​AGG​AGG​TCA​C
TGF-β1-R	AGA​GCA​ACA​CGG​GTT​CAG​GTA
Collagen I-F	CAA​TGC​TGC​CCT​TTC​TGC​TCC​TTT
Collagen I-R	ATT​GCC​TTT​GAT​TGC​TGG​GCA​GAC
Collagen III-F	GGT​CAC​TTT​CAC​TGG​TTG​ACG​A
Collagen III-R	TTG​AAT​ATC​AAA​CAC​GCA​AGG​C
GAPDH-F	AAG​AAG​GTG​GTG​AAG​CAG​GC
GAPDH-R	TCC​ACC​ACC​CTG​TTG​CTG​TA
U6-F	GCT​TCG​GCA​GCA​CAT​ATA​CTA​AAA​T
U6-R	CGC​TTC​ACG​AAT​TTG​CGT​GTC​AT

### Western Blot

Total tissue and cell proteins were extracted, and electrophoresis was performed on 10% sodium dodecyl sulfate acrylamide gels followed by protein transfer to nitrocellulose membranes. The membranes were then incubated overnight at 4°C with the following antibodies: caspase-1 (1:500), IL-1β (1:300), TGF-β1 (1:500), GSDMD (1:500), collagen I (1:500), collagen III (1:500) and GAPDH (1:1000) (ZSGB-BIO, China). Next day, the membranes were then incubated for 1 h with the corresponding secondary antibodies. Bands were imaged using a Odyssey Infrared Imaging System, and the grayscale values of the bands were determined using ImageJ software, with GAPDH was used as an internal control.

### Luciferase Assay

The 3′ untranslated region (3′-UTR) of caspase-1 with or without the miR-135b binding site was amplified and cloned into psi-CHECK2 as the wild-type (WT) and mutant (Mut) plasmids. Then, the WT or the Mut plasmids were co-transfected into rat cardiac fibroblasts together with 20 nM miR-NC or miR-135b using Lipofectamine 2000 (Invitrogen). Samples were assayed for luciferase activity after 48 h using Promega’s Dual-Luciferase Reporter Assay System (E1910).

### Data Analysis

The data are presented as the mean ± SD. Student’s *t*-test and one-way ANOVA were used to assess the differences between two groups or between multiple groups, respectively. Two-tailed *p* < 0.05 was considered statistically significant. The data were plotted using GraphPad Prism 7.0.

## Results

### Ranolazine Restrains Cardiac Dysfunction in Diabetic Rats

**FIGURE 1:**
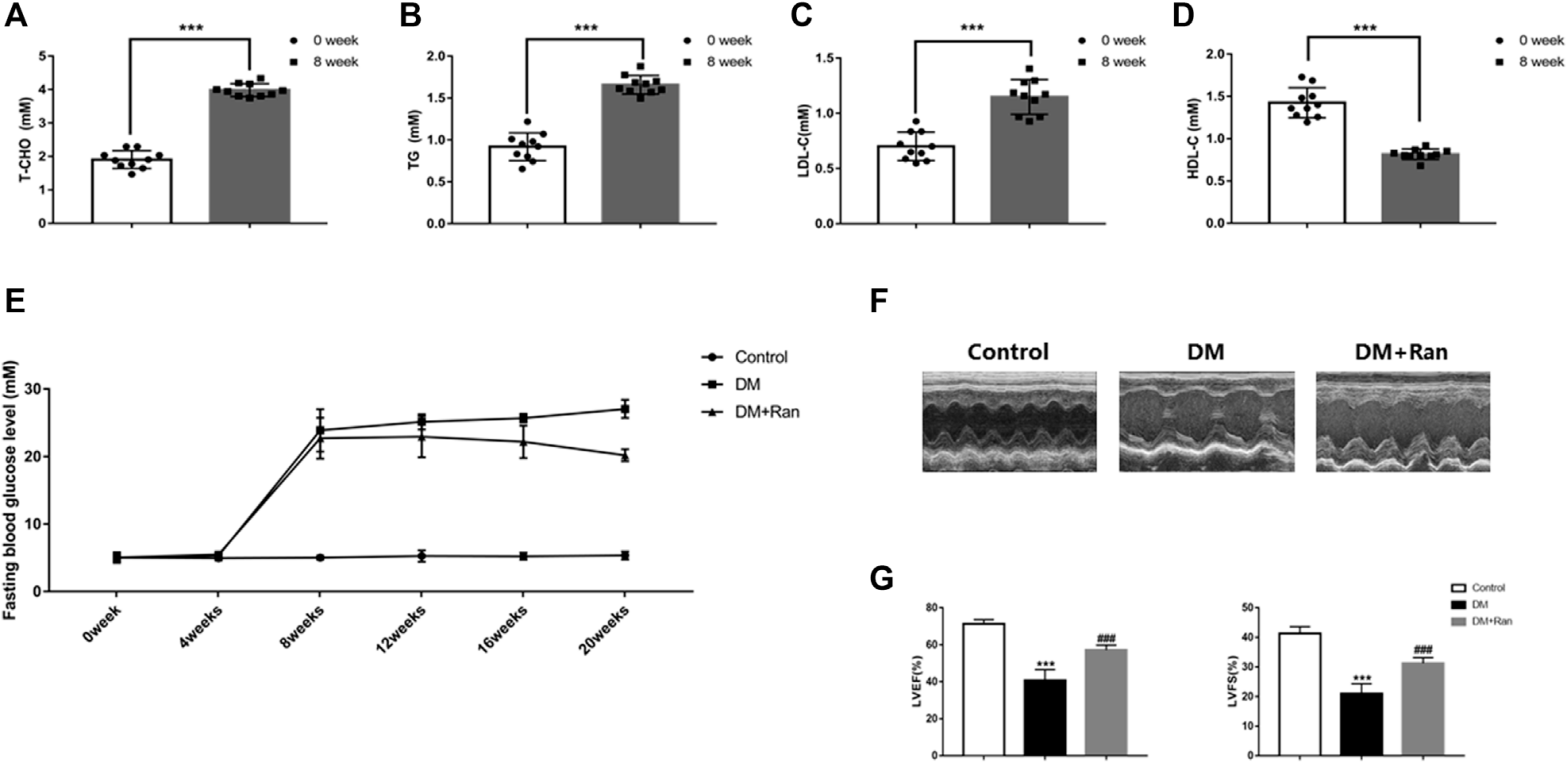
Ranolazine attenuates altered cardiac function in diabetic rats. **(A–D)** T-CHO, TG, LDL-C and HDL-C were measured at 0 and 8 weeks in animals fed a high-fat diet. **(E)** Changes in fasting blood glucose levels were assayed every 4 weeks in all groups of rats. **(F)** M-mode echocardiogram of the left ventricle. **(G)** The LVEF and LVFS of all groups are shown. ^***^ *p* < 0.001 *vs*. 0 weeks, ^***^ *p* < 0.001 *vs*. control, ^###^ *p* < 0.001 *vs*. DM; *n* = 6-10.

To validate the role of ranolazine in cardiac dysfunction in diabetic rats, we generated a model of type II diabetes in SD rats. After 8 weeks of feeding a high-fat diet, compared to the levels at baseline, T-CHO, TG and LDL-C were elevated, but HDL-C was significantly decreased (Figures 1A–D). We monitored fasting glucose every 4 weeks and found that the fasting glucose in the DM + Ran group was less than that in the DM group, but its value was still higher than that in the control group (Figure 1E). We performed echocardiographic measurements at 12 weeks after the diabetic model was established. The results showed that LVEF and LVFS were significantly lower in the DM group compared with the control group, while the impairment of cardiac function was reduced in the DM + Ran group compared with the DM group (Figures 1F,G). These results suggested that ranolazine inhibits cardiac dysfunction in diabetic rats.

### Ranolazine Suppresses Cardiac Fibrosis in Diabetic Rats

**FIGURE 2:**
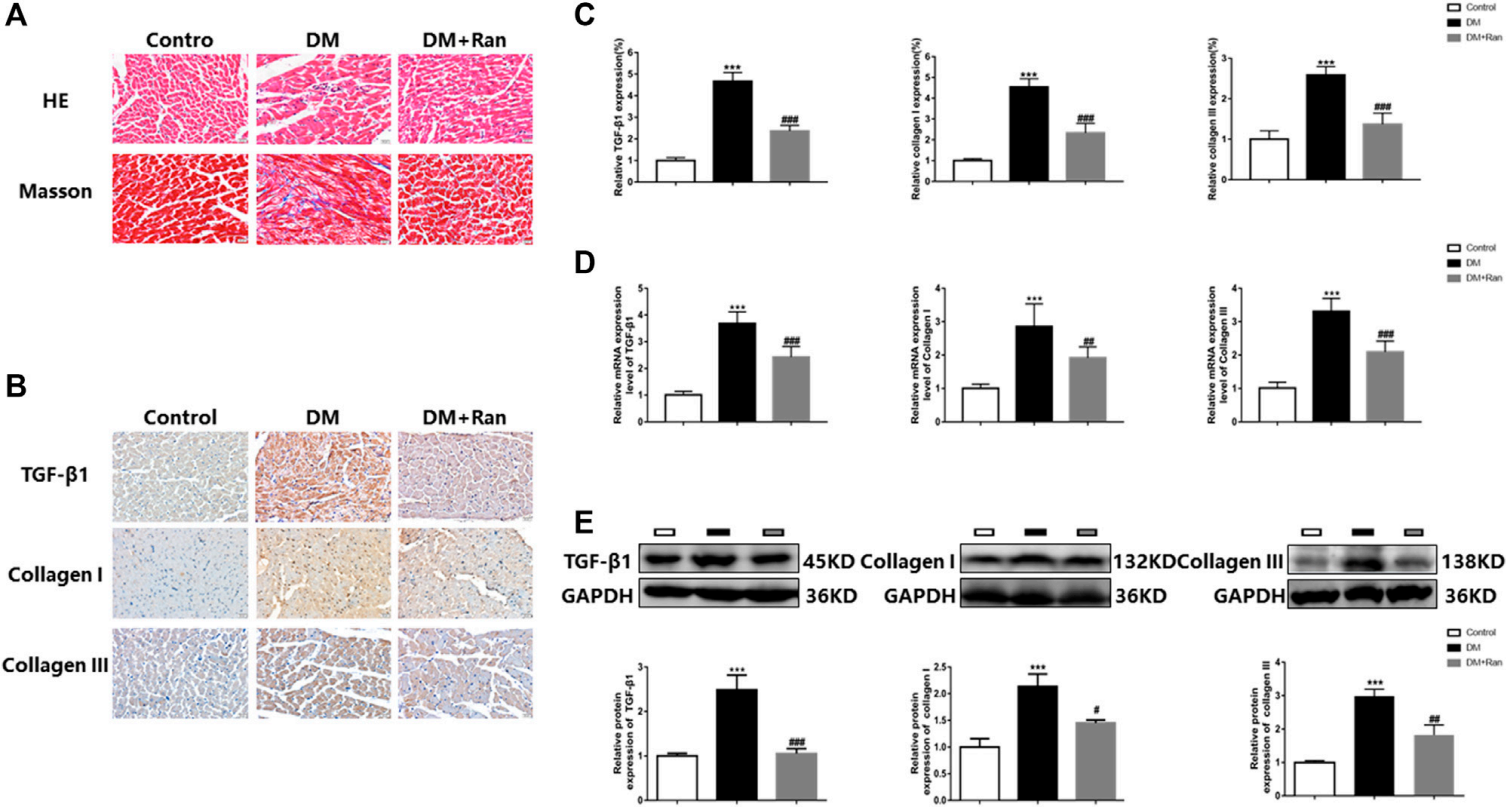
Ranolazine weakens cardiac fibrosis in diabetic rats. **(A)** Comparison of H&E and Masson staining among the groups. Scale bar = 20 μm. **(B,C)** Immunohistochemistry staining for TGF-β1, collagen I and collagen III. Scale bar = 20 μm. **(D)** Real-time PCR assay of the relative mRNA expression of TGF-β1, collagen I and collagen III. **(E)** Western blot assay of the relative protein expression of TGF-β1, collagen I and collagen III. ^***^ *p* < 0.001 *vs.* control, ^#^ *p* < 0.05 *vs*. DM, ^##^ *p* < 0.01 *vs.* DM, ^###^ *p* < 0.001 *vs.* DM; *n* = 6.

In view of the therapeutic function of ranolazine in the cardiac tissue of diabetic rats, we performed pathological tests on the cardiac tissue of diabetic rats. Morphological data obtained by H&E and Masson staining indicated that cardiac tissue in the DM group manifested inflammatory infiltration and higher collagen deposition, which were significantly suppressed in the DM + Ran group compared to those in the DM group (Figure 2A). The immunohistochemical analysis indicated an increase in the positive staining for TGF-β1, collagen I and collagen III in the DM group but a decrease in the positive staining for these factors after administration of ranolazine (Figures 2B,C). Additionally, the results indicated a significant increase in the mRNA levels of TGF-β1, collagen I and collagen III in the DM group compared to the control group, and the changes in the expression of these factors were reversed after administration of ranolazine (Figure 2D). In addition, the Western blot analysis results were consistent with the immunohistochemistry and mRNA data (Figure 2E). These results suggested that ranolazine reducs the occurrence of cardiac fibrosis in type II diabetic rats.

### Ranolazine Inhibits Pyroptosis in the Cardiac Tissue of Diabetic Rats

**FIGURE 3:**
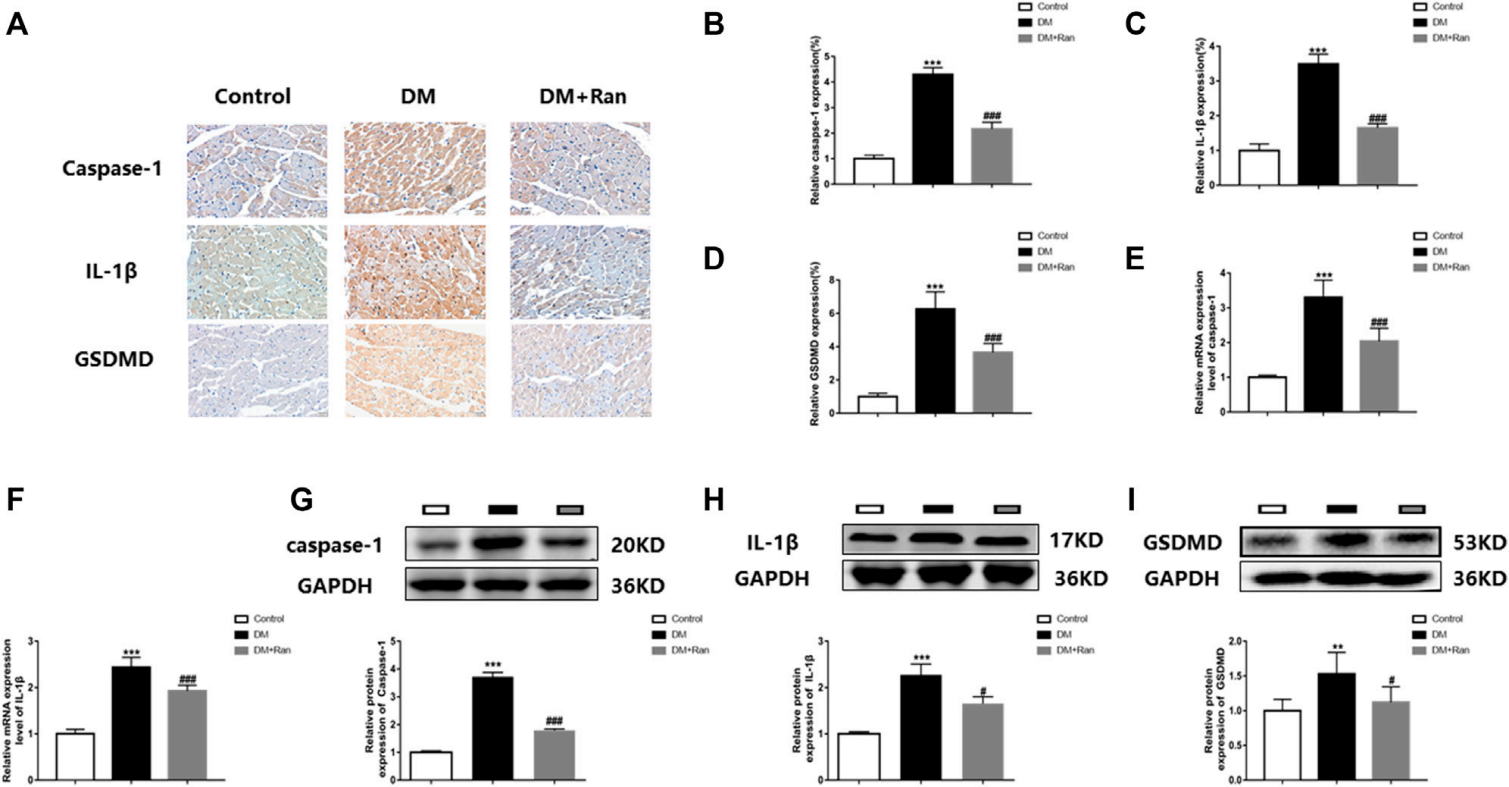
Ranolazine inhibits pyroptosis in diabetic rat cardiac tissues. **(A–D)** Immunohistochemical detection of caspase-1, IL-1β and GSDMD expression in cardiac tissues of rats in the groups. Scale bar = 20 μm. **(E,F)** Real-time PCR assay of relative mRNA expression of caspase-1 and IL-1β in cardiac tissues of rats in groups. **(G–I)** Western blot assay of relative protein expression of caspase-1, IL-1β and GSDMD proteins in cardiac tissues of rats in groups. ^**^ *p* < 0.01 *vs*. control, ^***^ *p* < 0.001 *vs*. control, ^#^ *p* < 0.05 *vs*. DM, ^###^ *p* < 0.001 *vs*. DM; *n* = 6.

Because pyroptosis promotes the expression of inflammatory factors that increase collagen deposition and fibrosis, we evaluated the expression of factors related to pyroptosis. Immunohistochemistry staining indicated that the expression of caspase-1, IL-1β and GSDMD was significantly increased in cardiac tissues of rats with DM, but decreased after ranolazine treatment (Figures 3A–D). The mRNA expression of caspase-1 and IL-1β was significantly increased in the DM group, but decreased after administration of ranolazine (Figures 3E,F). Moreover, Western blot analyses indicated that ranolazine reduced the expression of caspase-1, IL-1β and GSDMD (Figures 3G–I). These data suggested that ranolazine inhibits pyroptosis in cardiac tissue in diabetic rats.

### Ranolazine Inhibits Pyroptosis of Cardiac Fibroblasts *Via* miR-135b

**FIGURE 4:**
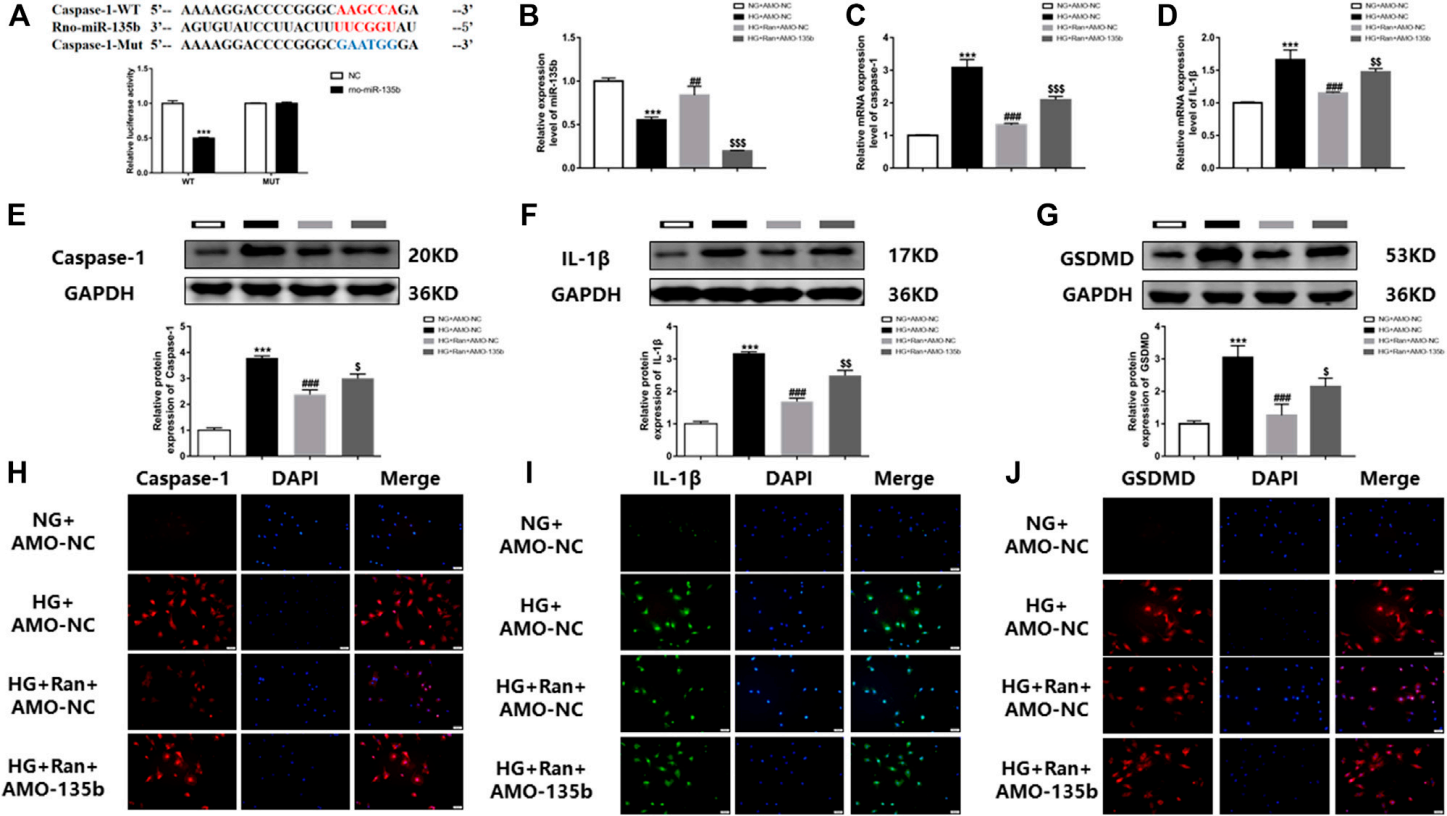
*In vitro* interference of miR-135b reduces ranolazine inhibition of cardiac fibroblast pyroptosis. **(A)** Binding targets between miR-135b and caspase-1. **(B)** Real-time PCR detection of changes relative to miR-135b expression in groups. **(C,D)** Real-time PCR was performed to detect the relative mRNA expression of caspase-1 and IL-1β in cardiac fibroblasts in the groups. **(E–G)** Western blot detection of the relative protein expression of caspase-1, IL-1β and GSDMD in cardiac fibroblasts. **(H–J)** Immunofluorescence detection of caspase-1, IL-1β and GSDMD expression in cardiac fibroblasts. Scale bar = 50 μm ^***^ *p* < 0.001 *vs.* NG + AMO-NC,^***^ *p* < 0.001 *vs*. NC, ^##^ *p* < 0.01 *vs*. HG + AMO-NC, ^###^ *p* < 0.001 *vs.* HG + AMO-NC, ^$^ *p* < 0.05 *vs*. HG + Ran + AMO-NC, ^$$^ *p* < 0.01 *vs.* HG + Ran + AMO-NC, ^$$$^ *p* < 0.001 *vs*. HG + Ran + AMO-NC; *n* = 3.

To demonstrate the involvement of miR-135b in the inhibition of pyroptosis of cardiac fibroblasts by ranolazine.The binding sequence of miR-135b to caspase-1 untranslated regions (3′UTRs) was predicted by bioinformatics (Figure 4A). Notably, a luciferase assay in rat cardiac fibroblasts confirmed the relationship between caspase-1 and miR-135b targeting (Figure 4A), changes in miR-135b expression were determined after high glucose stimulation of rat cardiac fibroblasts and treatment with ranolazine. The results showed that miR-135b expression was downregulated in the HG group, but increased following ranolazine treatment (Figure 4B). Subsequently, miR-135b expression was silenced in cells to detect changes in the expression of factors associated with pyroptosis, which revealed elevated mRNA and protein expression of caspase-1 and IL-1β as well as a similar trend for the protein expression of GSDMD (Figures 4C–G). In addition, the same results were obtained by immunofluorescence (Figures 4H–J). Together, these results suggested that ranolazine inhibits cardiac fibroblast pyroptosis *via* miR-135b.

### miR-135b Silencing Attenuates High Glucose-Induced Fibrosis in Fibroblasts Treated With Ranolazine

**FIGURE 5:**
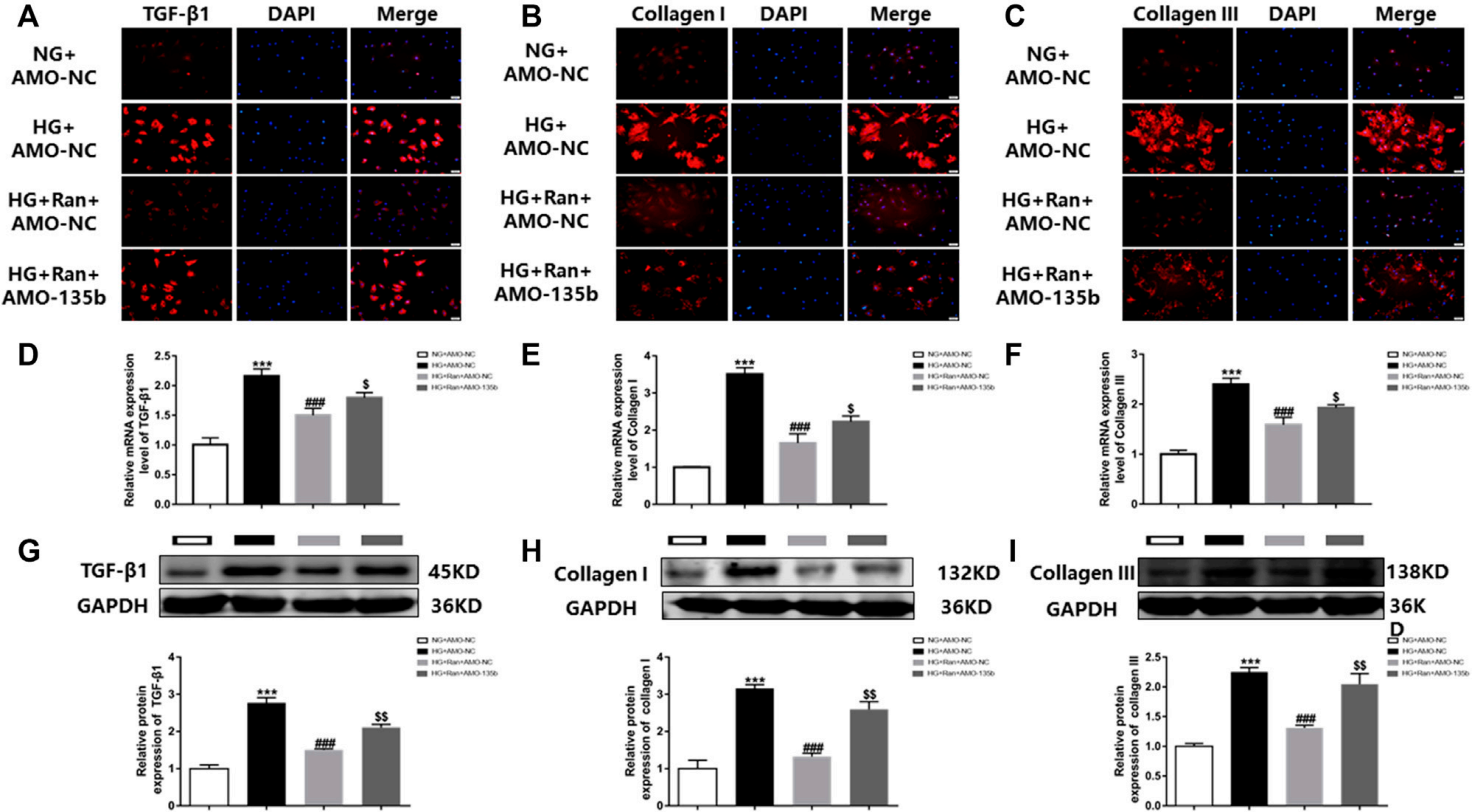
*In vitro* interference of miR-135b reduces ranolazine inhibition of cardiac fibrosis. **(A–C)** Immunofluorescence detection of TGF-β1, collagen I and collagen III expression in cardiac fibroblasts in the groups. **(D–F)** Real-time PCR was performed to detect the relative mRNA expression of TGF-β1, collagen I and collagen III in groups of cardiac fibroblasts. **(G–I)** Western blot analysis was performed to detect the relative protein expression of TGF-β1, collagen I and collagen III in cardiac fibroblasts. ^***^ *p* < 0.001 *vs.* NG + AMO-NC, ^###^ *p* < 0.001 *vs*. HG + AMO-NC, ^$^ *p* < 0.05 *vs*. HG + Ran + AMO-NC, ^$$^ *p* < 0.01 *vs*. HG + Ran + AMO-NC; *n* = 3.

The *in vivo* results indicated that ranolazine inhibited high glucose-induced cardiac fibrosis, which was verified *in vitro* by immunofluorescence. The data indicated that the fluorescence intensity of TGF-β1, collagen I and collagen III was increased in high glucose-stimulated cardiac fibroblasts but was significantly decreased after treatment with ranolazine. However, inhibition of fluorescence intensity was reduced after silencing miR-135b (Figures 5A–C). Moreover, RT–PCR analysis indicated that the mRNA levels of TGF-β1, collagen I and collagen III were increased in the HG group and decreased after treatment with ranolazine. In addition, miR-135b silencing did not reverse these changes induced by high glucose even after treatment with ranolazine (Figures 5D–F). In addition, the Western blot analysis results were similar to the immunofluorescence assay results (Figures 5G–I). These results suggested that ranolazine inhibition of high glucose-stimulated fibrosis of cardiac fibroblasts is achieved in coordination with miR-135b.

## Discussion

**FIGURE 6:**
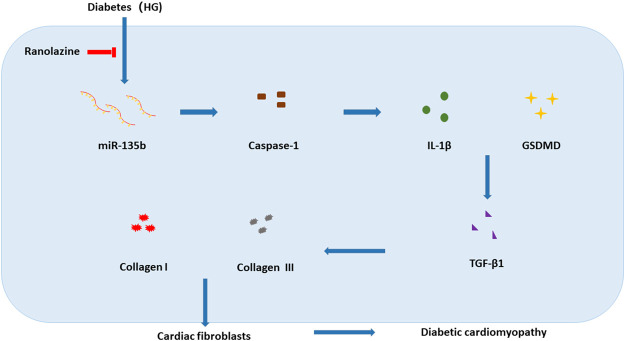
Model of ranolazine for the treatment of diabetic cardiac fibrosis by upregulating miR-135b to inhibit fibroblast pyroptosis and reduce collagen deposition.

DCM, a chronic disease with complex pathogenesis, has no clear clinical criteria for diagnosis. Patients with what was initially defined as DCM had pathological manifestations of cardiac fibrosis (Rubler et al., 1972); therefore, effective inhibition of cardiac fibrosis may help to treat DCM. In the present study, we verified that ranolazine inhibited diabetic cardiac fibrosis and that miR-135b reduced collagen deposition by inhibiting cardiac fibroblast pyroptosis. We elucidated for the first time the involvement of miR-135b inhibition of pyroptosis in the treatment of diabetic cardiac fibrosis with ranolazine (Figure 6).

Ranolazine is recommended as a second-line agent for chronic stable angina pectoris by European and North American clinical guidelines (Task Force et al., 2013). Ranolazine has a cardioprotective effect and is particularly effective in functional restoration after myocardial infarction in patients with diabetes (Odiete et al., 2013). Persistent hyperglycemia is a major cause of cardiovascular complications in diabetic patients. Ranolazine lowers the levels of glycated hemoglobin and fasting blood glucose, and it inhibits the release of glucagon in diabetic patients, which has a positive effect on the treatment of diabetes (Chisholm et al., 2010; Dhalla et al., 2014; Eckel et al., 2015). Moreover, Ranolazine has a protective effect on cardiomyocytes stimulated by high glucose; however, the effect on fibroblasts has not been elucidated (Chen et al., 2020). The results of the present study indicated that ranolazine attenuated cardiac inflammation and collagen deposition. Moreover, ranolazine restrained cardiac fibrosis and improved cardiac dysfunction and reconstruction in DM rats by inhibiting the expression of collagen I and collagen III.

miRNA-135b is closely associated with a variety of diseases, including cancer, heart disease and epilepsy (Umezu et al., 2014). Recently, miR-135b has been reported to promote M2 polarization of synovial macrophages by targeting MAPK6, thereby reducing cartilage damage (Wang and Xu, 2021). miR-135b inhibits the immune response of Th17 cells by targeting CXCL12, indicating the potential value of miR-135b in the treatment of inflammation (Liu et al., 2020). However, relationships between miR-135b and DCM have not been reported. A decrease in the expression of miR-135b in retinal cells exposed to high glucose levels suggests a relationship between miR-135b and diabetic complications (Gong et al., 2017). The present study demonstrated that miR-135b expression was reduced in high glucose-stimulated cardiac fibroblasts and that ranolazine induced an increase in miR-135b expression. Interference with miR-135b had little effect on the expression of downstream factors even after treatment with ranolazine, suggesting that the effects of ranolazine in diabetic cardiac fibrosis are mediated by upregulation of miR-135b. The present study is the first to demonstrate differential expression of miR-135b in a model of DCM and to show that ranolazine modulates miR-135b expression in diabetic cardiac fibrosis.

Pyroptosis is involved in the development and progression of DCM (Yang et al., 2018). Caspase-1 plays an important role in the production of pyroptosis and the release of related factors (Miao et al., 2010). The present study demonstrated that caspase-1 expression was increased in high glucose-stimulated cardiac fibroblasts and diabetic rats, which led to promotion of downstream production of IL-1β and GSDMD. GSDMD expression is closely associated with cell pyroptosis (Kovacs and Miao, 2017). At the same time, prolonged exposure of microvascular endothelial cells to IL-1β activates the TGF-β1 signaling pathway, which induces the conversion of microvascular endothelial cells into fibroblasts and increases collagen deposition, ultimately leading to cardiac remodeling and the generation of myocardial interstitial fibrosis (Fernandez and Mosquera, 2002; Artlett, 2012; Wang et al., 2013). In *in vitro* and *in vivo* experiments, activation of pyroptosis was found to have triggered the expression of the downstream factors, including TGF-β1, collagen I and collagen III. Thus, we concluded that miR-135b inhibits pyroptosis of cardiac fibroblasts and reduces collagen deposition.

Although we demonstrated the protective effect of ranolazine on high glucose-stimulated cardiac fibroblasts, our experiments had several limitations. First, we only demonstrated *in vitro* that ranolazine reduces the damage of cardiac fibroblasts induced by high glucose by regulating miR-135b, and it remains unclear whether the same results can be obtained with *in vivo* experiments. Second, ranolazine protects cardiac fibroblasts by reducing caspase-1-mediated pyroptosis, but it needs to be further verified whether the same results can be obtained *in vivo* or by human-specific knockdown of caspase-1.

In conclusion, we validated the effect of ranolazine on diabetic cardiac fibrosis, and we reported for the first time that hyperglycemia decreases miR-135b expression in cardiac fibroblasts and that ranolazine reverses this change. Ranolazine treatment of diabetic cardiac fibrosis is mediated by the miR-135b/caspase-1/TGF-β1 pathway. Our study provides a solid theoretical basis for understanding the pathogenesis of diabetic cardiac fibrosis and the clinical use of ranolazine in the treatment of DCM.

## Data Availability

The original contributions presented in the study are included in the article/Supplementary Material, further inquiries can be directed to the corresponding author.

## References

[B1] Artlett C. M. (2012). The Role of the NLRP3 Inflammasome in Fibrosis. Open. Rheumatol. J. 6, 80–86. 10.2174/1874312901206010080 22802905 PMC3395884

[B2] Asbun J. Villarreal F. J. (2006). The Pathogenesis of Myocardial Fibrosis in the Setting of Diabetic Cardiomyopathy. J. Am. Coll. Cardiol. 47, 693–700. 10.1016/j.jacc.2005.09.050 16487830

[B3] Banerjee K. Ghosh R. K. Kamatam S. Banerjee A. Gupta A. (2017). Role of Ranolazine in Cardiovascular Disease and Diabetes: Exploring beyond Angina. Int. J. Cardiol. 227, 556–564. 10.1016/j.ijcard.2016.10.102 27838121

[B4] Bergsbaken T. Fink S. L. Cookson B. T. (2009). Pyroptosis: Host Cell Death and Inflammation. Nat. Rev. Microbiol. 7, 99–109. 10.1038/nrmicro2070 19148178 PMC2910423

[B5] Bracey N. A. Gershkovich B. Chun J. Vilaysane A. Meijndert H. C. Wright J. R. JR. (2014). Mitochondrial NLRP3 Protein Induces Reactive Oxygen Species to Promote Smad Protein Signaling and Fibrosis Independent from the Inflammasome. J. Biol. Chem. 289, 19571–19584. 10.1074/jbc.M114.550624 24841199 PMC4094069

[B6] Cappetta D. Esposito G. Coppini R. Piegari E. Russo R. Ciuffreda L. P. (2017). Effects of Ranolazine in a Model of Doxorubicin-Induced Left Ventricle Diastolic Dysfunction. Br. J. Pharmacol. 174, 3696–3712. 10.1111/bph.13791 28320043 PMC5647186

[B7] Chen X. Ren L. Liu X. Sun X. Dong C. Jiang Y. (2020). Ranolazine Protects against Diabetic Cardiomyopathy by Activating the NOTCH1/NRG1 Pathway. Life Sci. 261, 118306. 10.1016/j.lfs.2020.118306 32828943

[B8] Chisholm J. W. Goldfine A. B. Dhalla A. K. Braunwald E. Morrow D. A. Karwatowska-Prokopczuk E. (2010). Effect of Ranolazine on A1C and Glucose Levels in Hyperglycemic Patients with Non-ST Elevation Acute Coronary Syndrome. Diabetes Care 33, 1163–1168. 10.2337/dc09-2334 20357382 PMC2875416

[B9] Cho N. H. Shaw J. E. Karuranga S. Huang Y. Da Rocha Fernandes J. D. Ohlrogge A. W. (2018). IDF Diabetes Atlas: Global Estimates of Diabetes Prevalence for 2017 and Projections for 2045. Diabetes Res. Clin. Pract. 138, 271–281. 10.1016/j.diabres.2018.02.023 29496507

[B10] Chu Q. Li A. Chen X. Qin Y. Sun X. Li Y. (2018). Overexpression of miR-135b Attenuates Pathological Cardiac Hypertrophy by Targeting CACNA1C. Int. J. Cardiol. 269, 235–241. 10.1016/j.ijcard.2018.07.016 30037628

[B11] de Simone G. Devereux R. B. Chinali M. Lee E. T. Galloway J. M. Barac A. (2010). Diabetes and Incident Heart Failure in Hypertensive and Normotensive Participants of the Strong Heart Study. J. Hypertens. 28, 353–360. 10.1097/HJH.0b013e3283331169 19844184 PMC3005764

[B12] Dhalla A. K. Yang M. Ning Y. Kahlig K. M. Krause M. Rajamani S. (2014). Blockade of Na+ Channels in Pancreatic α-Cells Has Antidiabetic Effects. Diabetes 63, 3545–3556. 10.2337/db13-1562 24812428

[B13] Eckel R. H. Henry R. R. Yue P. Dhalla A. Wong P. Jochelson P. (2015). Effect of Ranolazine Monotherapy on Glycemic Control in Subjects with Type 2 Diabetes. Diabetes Care 38, 1189–1196. 10.2337/dc14-2629 26049552 PMC4477340

[B14] Fernández L. Mosquera J. A. (2002). Interleukin-1 Increases Fibronectin Production by Cultured Rat Cardiac Fibroblasts. Pathobiology 70, 191–196. 10.1159/000069328 12679595

[B15] Gong Q. Xie J. n. Liu Y. Li Y. Su G. (2017). Differentially Expressed MicroRNAs in the Development of Early Diabetic Retinopathy. J. Diabetes Res. 2017, 1–10. 10.1155/2017/4727942 PMC5494571 28706953

[B16] Guariguata L. Whiting D. R. Hambleton I. Beagley J. Linnenkamp U. Shaw J. E. (2014). Global Estimates of Diabetes Prevalence for 2013 and Projections for 2035. Diabetes Res. Clin. Pract. 103, 137–149. 10.1016/j.diabres.2013.11.002 24630390

[B17] Hölscher M. Bode C. Bugger H. (2016). Diabetic Cardiomyopathy: Does the Type of Diabetes Matter? Int. J. Mol. Sci. 17, 2136. 10.3390/ijms17122136 PMC5187936 27999359

[B18] Kovacs S. B. Miao E. A. (2017). Gasdermins: Effectors of Pyroptosis. Trends Cell Biol. 27, 673–684. 10.1016/j.tcb.2017.05.005 28619472 PMC5565696

[B19] Li A. Yu Y. Ding X. Qin Y. Jiang Y. Wang X. (2020). MiR-135b Protects Cardiomyocytes from Infarction through Restraining the NLRP3/caspase-1/IL-1β Pathway. Int. J. Cardiol. 307, 137–145. 10.1016/j.ijcard.2019.09.055 31870781

[B20] Li X. du N. Zhang Q. Li J. Chen X. Liu X. (2014). MicroRNA-30d Regulates Cardiomyocyte Pyroptosis by Directly Targeting Foxo3a in Diabetic Cardiomyopathy. Cell Death Dis 5, e1479. 10.1038/cddis.2014.430 25341033 PMC4237254

[B21] Liu Y. Huo S.-G. Xu L. Che Y.-Y. Jiang S.-Y. Zhu L. (2020). MiR-135b Alleviates Airway Inflammation in Asthmatic Children and Experimental Mice with Asthma via Regulating CXCL12. Immunological Invest., 1–15. 10.1080/08820139.2020.1841221 33203292

[B22] Mao Q. Liang X.-L. Zhang C.-L. Pang Y.-H. Lu Y.-X. (2019). LncRNA KLF3-AS1 in Human Mesenchymal Stem Cell-Derived Exosomes Ameliorates Pyroptosis of Cardiomyocytes and Myocardial Infarction through miR-138-5p/Sirt1 axis. Stem Cell Res Ther 10, 393. 10.1186/s13287-019-1522-4 31847890 PMC6918658

[B23] Miao E. A. Leaf I. A. Treuting P. M. Mao D. P. Dors M. Sarkar A. (2010). Caspase-1-induced Pyroptosis Is an Innate Immune Effector Mechanism against Intracellular Bacteria. Nat. Immunol. 11, 1136–1142. 10.1038/ni.1960 21057511 PMC3058225

[B24] Odiete O. Konik E. A. Sawyer D. B. Hill M. F. (2013). Type 1 Diabetes Mellitus Abrogates Compensatory Augmentation of Myocardial neuregulin-1β/ErbB in Response to Myocardial Infarction Resulting in Worsening Heart Failure. Cardiovasc. Diabetol. 12, 52. 10.1186/1475-2840-12-52 23530877 PMC3617023

[B25] Palomer X. Román-Azcona M. S. Pizarro-Delgado J. Planavila A. Villarroya F. Valenzuela-Alcaraz B. (2020). SIRT3-mediated Inhibition of FOS through Histone H3 Deacetylation Prevents Cardiac Fibrosis and Inflammation. Sig Transduct Target. Ther. 5, 14. 10.1038/s41392-020-0114-1 PMC7046732 32296036

[B26] Rubler S. Dlugash J. Yuceoglu Y. Z. Kumral T. Branwood A. W. Grishman A. (1972). New Type of Cardiomyopathy Associated with Diabetic Glomerulosclerosis. Am. J. Cardiol. 30, 595–602. 10.1016/0002-9149(72)90595-4 4263660

[B27] Schroder K. Zhou R. Tschopp J. (2010). The NLRP3 Inflammasome: a Sensor for Metabolic Danger. Science 327, 296–300. 10.1126/science.1184003 20075245

[B28] Task Force M. Montalescot G. Sechtem U. Achenbach S. Andreotti F. Arden C. (2013). 2013 ESC Guidelines on the Management of Stable Coronary Artery Disease. Eur. Heart J. 34, 2949–3003. 10.1093/eurheartj/eht296 23996286

[B29] Umezu T. Tadokoro H. Azuma K. Yoshizawa S. Ohyashiki K. Ohyashiki J. H. (2014). Exosomal miR-135b Shed from Hypoxic Multiple Myeloma Cells Enhances Angiogenesis by Targeting Factor-Inhibiting HIF-1. Blood 124, 3748–3757. 10.1182/blood-2014-05-576116 25320245 PMC4263983

[B30] Wang G. T. Li H. Yu Z. Q. He X. N. (2019). Effects of Ranolazine on Cardiac Function in Rats with Heart Failure. Eur. Rev. Med. Pharmacol. Sci. 23, 9625–9632. 10.26355/eurrev_201911_19456 31773713

[B31] Wang H. Lu Y. Yan Y. Tian S. Zheng D. Leng D. (2019). Promising Treatment for Type 2 Diabetes: Fecal Microbiota Transplantation Reverses Insulin Resistance and Impaired Islets. Front. Cel. Infect. Microbiol. 9, 455. 10.3389/fcimb.2019.00455 PMC6979041 32010641

[B32] Wang R. Xu B. (2021). TGF-β1-modified MSC-Derived Exosomal miR-135b Attenuates Cartilage Injury via Promoting M2 Synovial Macrophage Polarization by Targeting MAPK6. Cell Tissue Res 384, 113–127. 10.1007/s00441-020-03319-1 33404840

[B33] Wang Y. Wu Y. Chen J. Zhao S. Li H. (2013). Pirfenidone Attenuates Cardiac Fibrosis in a Mouse Model of TAC-Induced Left Ventricular Remodeling by Suppressing NLRP3 Inflammasome Formation. Cardiology 126, 1–11. 10.1159/000351179 23839341

[B34] Yang F. Li A. Qin Y. Che H. Wang Y. Lv J. (2019). A Novel Circular RNA Mediates Pyroptosis of Diabetic Cardiomyopathy by Functioning as a Competing Endogenous RNA. Mol. Ther. - Nucleic Acids 17, 636–643. 10.1016/j.omtn.2019.06.026 31400606 PMC6700436

[B35] Yang F. Qin Y. Lv J. Wang Y. Che H. Chen X. (2018). Silencing Long Non-coding RNA Kcnq1ot1 Alleviates Pyroptosis and Fibrosis in Diabetic Cardiomyopathy. Cell Death Dis 9, 1000. 10.1038/s41419-018-1029-4 30250027 PMC6155223

