# Supplementary material for: Ranolazine Inhibits Pyroptosis via Regulation of miR-135b in the Treatment of Diabetic Cardiac Fibrosis
Source: Front Mol Biosci. 2022 Jan 26;9:806966. doi: 10.3389/fmolb.2022.806966 (PMC8826643; doi:10.3389/fmolb.2022.806966)

Figure1A

	Sample	Blank	Standard	
0weeks-1	0.129	0.05	0.250666667	
0weeks-2	0.117			
0weeks-3	0.129			
0weeks-4	0.139			
0weeks-5	0.139			Standard concentration=5.17 mM
0weeks-6	0.124			
0weeks-7	0.123			
0weeks-8	0.107			
0weeks-9	0.114			
0weeks-10	0.119			

	Sample	Blank	Standard	
8weeks-1	0.187	0.050333333	0.236	
8weeks-2	0.194			
8weeks-3	0.2			
8weeks-4	0.206			
8weeks-5	0.201			Standard concentration=5.17 mM
8weeks-6	0.193			
8weeks-7	0.187			
8weeks-8	0.189			
8weeks-9	0.185			
8weeks-10	0.192			

Figure1B

	Sample	Blank	Standard
0weeks-1	0.083	0.047	0.123
0weeks-2	0.072		
0weeks-3	0.08		
0weeks-4	0.078		
0weeks-5	0.081		
0weeks-6	0.079		
0weeks-7	0.088		
0weeks-8	0.069		
0weeks-9	0.074		
0weeks-10	0.075		

Standard concentration=2.26 mM

	Sample	Blank	Standard
8week-1	0.174	0.046	0.2
8week-2	0.162		
8week-3	0.161		
8week-4	0.154		
8week-5	0.155		
8week-6	0.153		
8week-7	0.156		
8week-8	0.148		
8week-9	0.16		
8week-10	0.167		

Standard concentration=2.26 mM

Figure1C

	Sample1	Sample2	Blank1	Blank2	Standard1	Standard2
0weeks-1	0.079	0.137	0.048	0.051	0.062	0.312333333
0weeks-2	0.076	0.136				
0weeks-3	0.078	0.149				
0weeks-4	0.068	0.133				
0weeks-5	0.066	0.15				
0weeks-6	0.076	0.132				
0weeks-7	0.082	0.148	Standard concentration=2.55mM			
0weeks-8	0.081	0.154				
0weeks-9	0.073	0.166				
0weeks-10	0.065	0.149				

	Sample1	Sample2	Blank1	Blank2	Standard1	Standard2
8weeks-1	0.058	0.157	0.044333333	0.056	0.065666667	0.265
8weeks-2	0.059	0.142				
8weeks-3	0.058	0.152				
8weeks-4	0.058	0.141				
8weeks-5	0.052	0.158	Standard concentration=2.55mM			
8weeks-6	0.055	0.153				
8weeks-7	0.054	0.169				
8weeks-8	0.052	0.159				
8weeks-9	0.057	0.154				
8weeks-10	0.059	0.139				

Figure1D

	Sample1	Sample2	Blank1	Blank2	Standard1	Standard2
0week-1	0.057	0.123	0.047333333	0.052	0.057666667	0.116
0week-2	0.062	0.125				
0week-3	0.06	0.131				
0week-4	0.055	0.122				
0week-5	0.054	0.141				
0week-6	0.062	0.14				
0week-7	0.066	0.137	Standard concentration=1.1 mM			
0week-8	0.063	0.136				
0week-9	0.066	0.143				
0week-10	0.052	0.141				

	Sample1	Sample2	Blank1	Blank2	Standard1	Standard2
8weeks-1	0.052	0.157	0.041666667	0.049	0.062333333	0.203333333
8weeks-2	0.05	0.158				
8weeks-3	0.055	0.161				
8weeks-4	0.053	0.172				
8weeks-5	0.059	0.149				
8weeks-6	0.058	0.162	Standard concentration=1.1 mM			
8weeks-7	0.052	0.163				
8weeks-8	0.054	0.167				
8weeks-9	0.057	0.161				
8weeks-1(	0.058	0.163				

Figure1E

Fasting blood glucose data						
	0weeks	4weeks	8weeks	12weeks	16weeks	20weeks
Control	5.6	4.9	5.5	6.5	5.8	5.6
	4.7	5.2	4.9	4.7	4.7	5.8
	5.2	4.7	4.8	5.8	5.2	5.6
	4.9	5.5	4.7	4.2	4.6	4.5
	5.3	4.6	5.2	4.9	5.7	5.9
	4.6	4.8	5.1	5.6	5.5	4.7
DM	4.3	5.6	22.1	23.5	25.5	26.5
	4.8	5.8	22.3	24.7	24.6	24.9
	4.6	4.6	19.8	25.6	26.3	27.5
	5.3	5.7	25.7	24.7	25.9	28.4
	6.1	4.6	24.9	26.8	26.4	26.7
	4.9	5.7	28.6	25.7	25.6	28.4
DM+Ran 30mg/Kg	5.8	5.6	25.7	24.7	21.7	20.6
	6.3	5.9	23.6	25.8	20.5	19.8
	4.7	5.4	23.7	23.4	26.7	20.6
	4.4	5.5	18.6	19.8	19.9	21.5
	4.6	5.6	19.4	18.6	21.6	18.9
	4.5	5.1	25.4	25.4	22.8	19.9

Figure1F

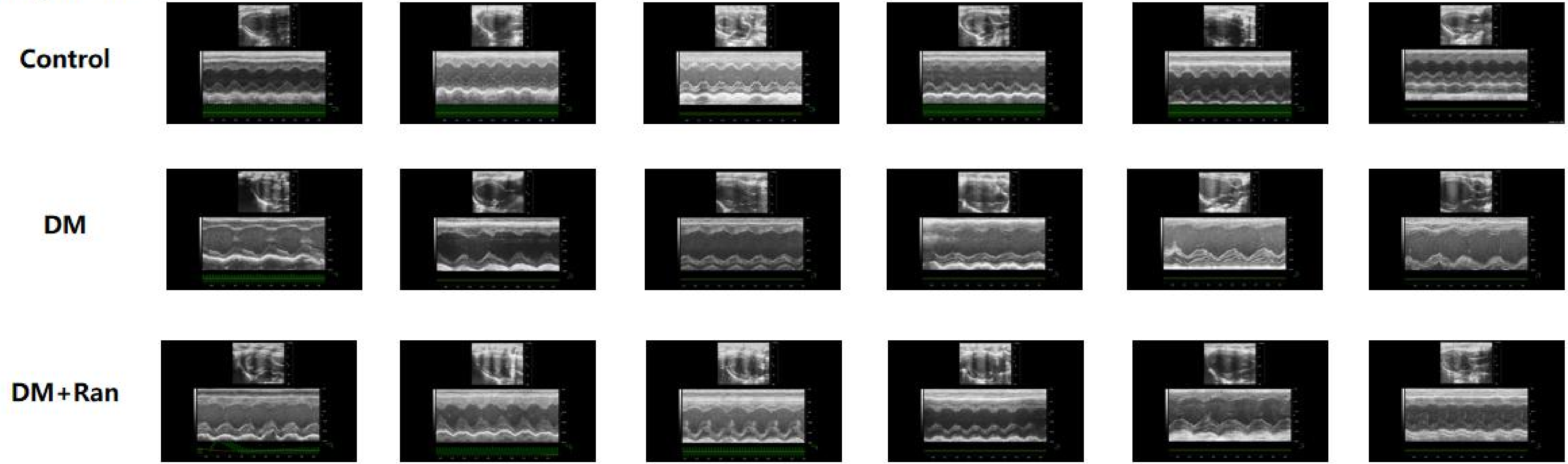

Figure1G

LVEF				LVFS			
	Control	DM	DM+Ran		Control	DM	DM+Ran
	69.146013	47.601025	52.997376		39.246116	24.999997	28.097723
	68.983898	36.753855	58.831105		38.642299	18.5079	32.028464
	73.892656	46.885832	59.308234		43.689314	24.474471	32.913174
	73.995943	42.881029	55.329503		43.595034	22.058816	29.969888
	69.83189	34.191186	59.944578		40.264649	17.282481	33.125002
	71.812096	36.572498	56.519654		42.647051	18.32061	31.301285

Figure2A

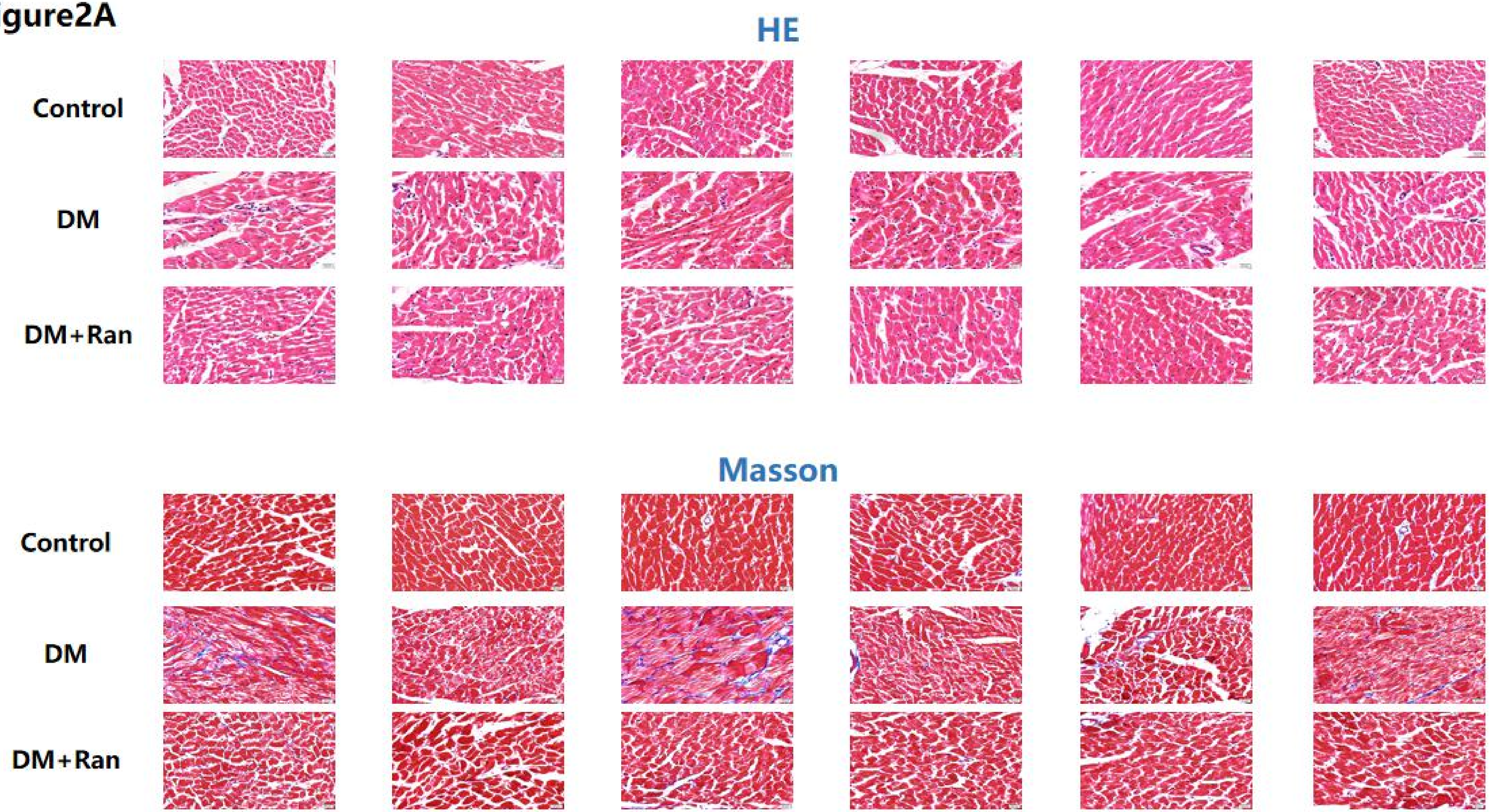

Figure2B

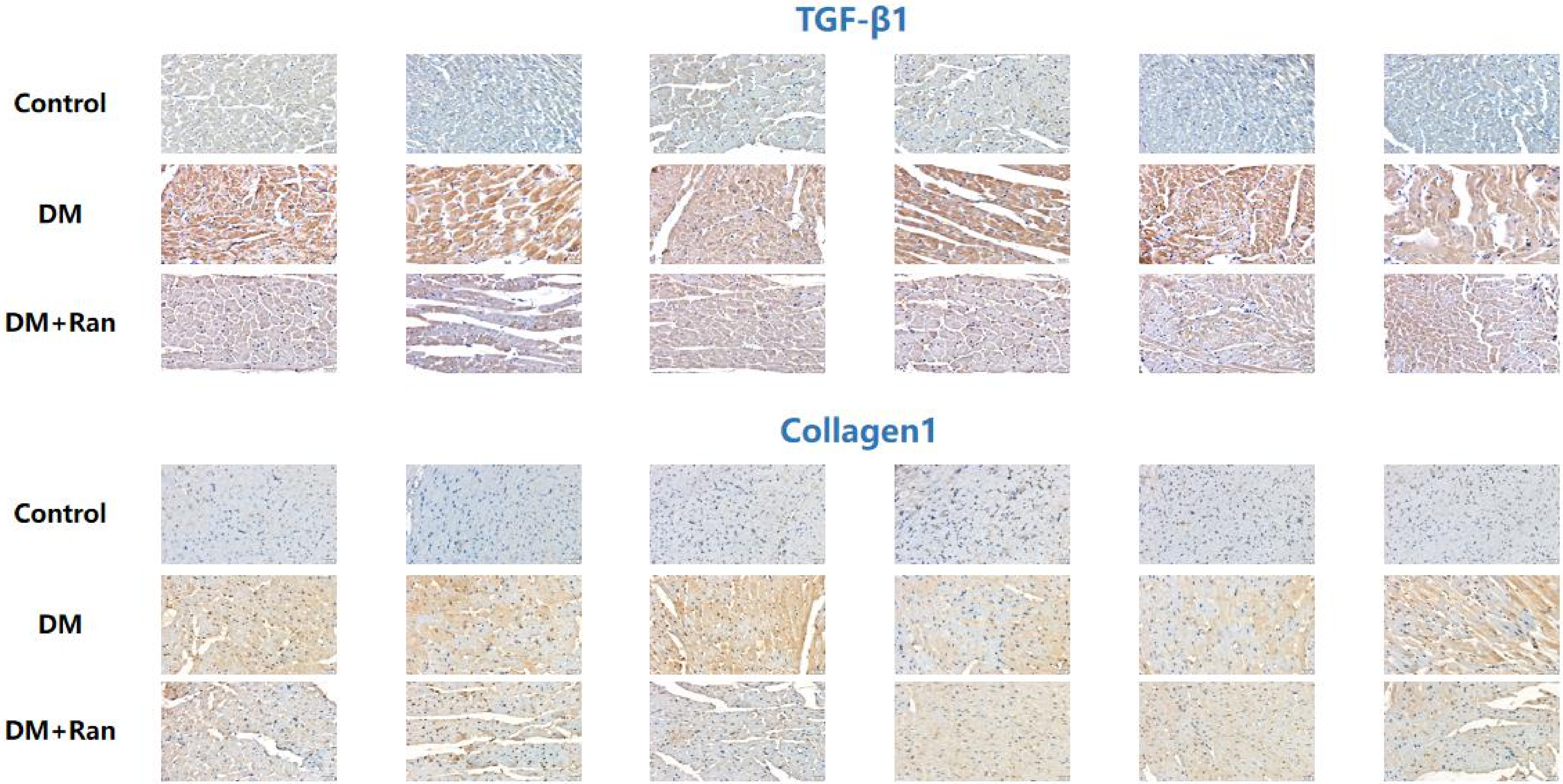

Figure2B

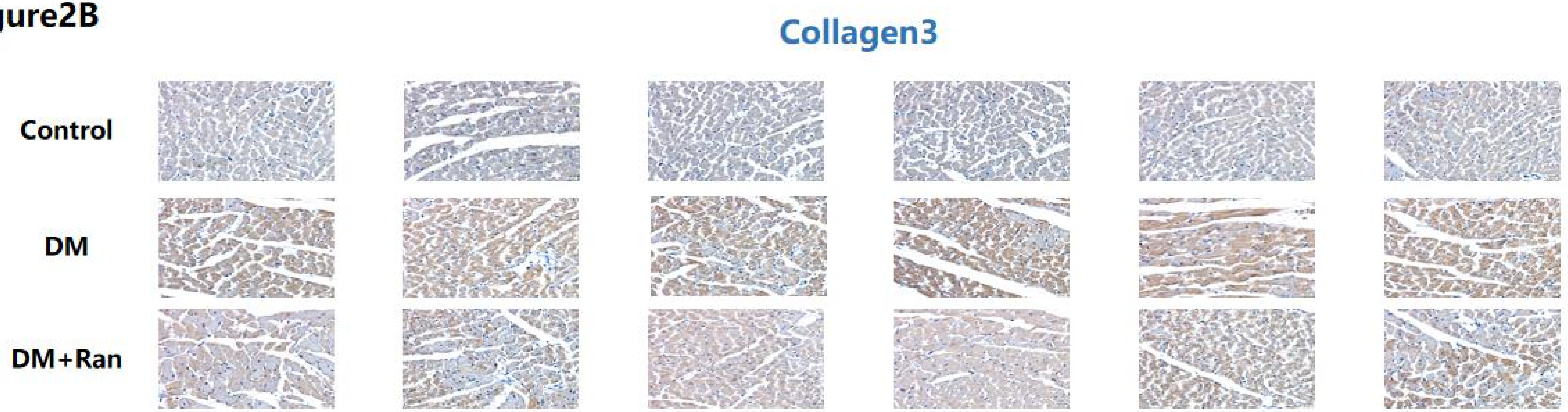

Figure2C

	TGF-β1	Collagen1	Collagen3
Control-1	7.71	6.05	8.59
Control-2	8.23	5.51	9.48
Control-3	6.05	4.93	14.43
Control-4	8.27	6.36	13.61
Control-5	8.96	5.39	14.22
Control-6	7.87	6.13	11.82
DM-1	37.65	24.48	34.56
DM-2	39.85	28.56	32.56
DM-3	38.43	24.94	31.99
DM-4	36.65	25.34	28.42
DM-5	36.70	23.59	28.05
DM-6	30.56	29.09	31.14
DM+Ran-1	18.25	13.57	14.29
DM+Ran-2	16.61	11.61	16.12
DM+Ran-3	17.2	15.36	21.11
DM+Ran-4	17.86	11.64	12.91
DM+Ran-5	22.28	10.60	19.97
DM+Ran-6	19.16	17.47	14.46

Figure2D

	GAPDH	TGF-β1
Control-1	14.51	26.92
Control-2	14.69	26.95
Control-3	15.29	27.34
Control-4	15.34	27.94
Control-5	15.22	27.58
Control-6	15.74	28.18
DM-1	14.73	25.4
DM-2	15.49	25.83
DM-3	14.59	25.01
DM-4	15.73	26.29
DM-5	15.86	26.11
DM-6	14.34	24.99
DM+Ran-1	15.29	26.26
DM+Ran-2	15.64	26.63
DM+Ran-3	14.51	25.55
DM+Ran-4	14.56	25.64
DM+Ran-5	14.72	25.59
DM+Ran-6	14.83	26.45

	GAPDH	Collagen1
Control-1	15.27	26.73
Control-2	15.13	26.57
Control-3	15.29	26.49
Control-4	15.34	26.38
Control-5	14.64	25.75
Control-6	14.27	25.39
DM-1	14.73	24.84
DM-2	14.68	24.52
DM-3	15.86	25.34
DM-4	14.73	23.98
DM-5	15.86	25.61
DM-6	14.34	24.39
DM+Ran-1	15.29	25.55
DM+Ran-2	14.44	24.84
DM+Ran-3	14.51	24.91
DM+Ran-4	14.56	24.87
DM+Ran-5	14.72	24.61
DM+Ran-6	15.36	25.94

	GAPDH	Collagen3
Control-1	14.51	28.55
Control-2	15.13	29.75
Control-3	15.29	29.37
Control-4	15.34	29.28
Control-5	15.22	29.64
Control-6	14.27	28.49
DM-1	14.73	27.14
DM-2	15.49	27.88
DM-3	14.59	27.27
DM-4	15.73	28.04
DM-5	15.86	28.33
DM-6	15.47	28.23
DM+Ran-1	14.29	27.76
DM+Ran-2	14.44	27.53
DM+Ran-3	14.51	27.72
DM+Ran-4	14.56	27.36
DM+Ran-5	15.94	29.19
DM+Ran-6	15.36	28.56

Figure2E-TGF-β1

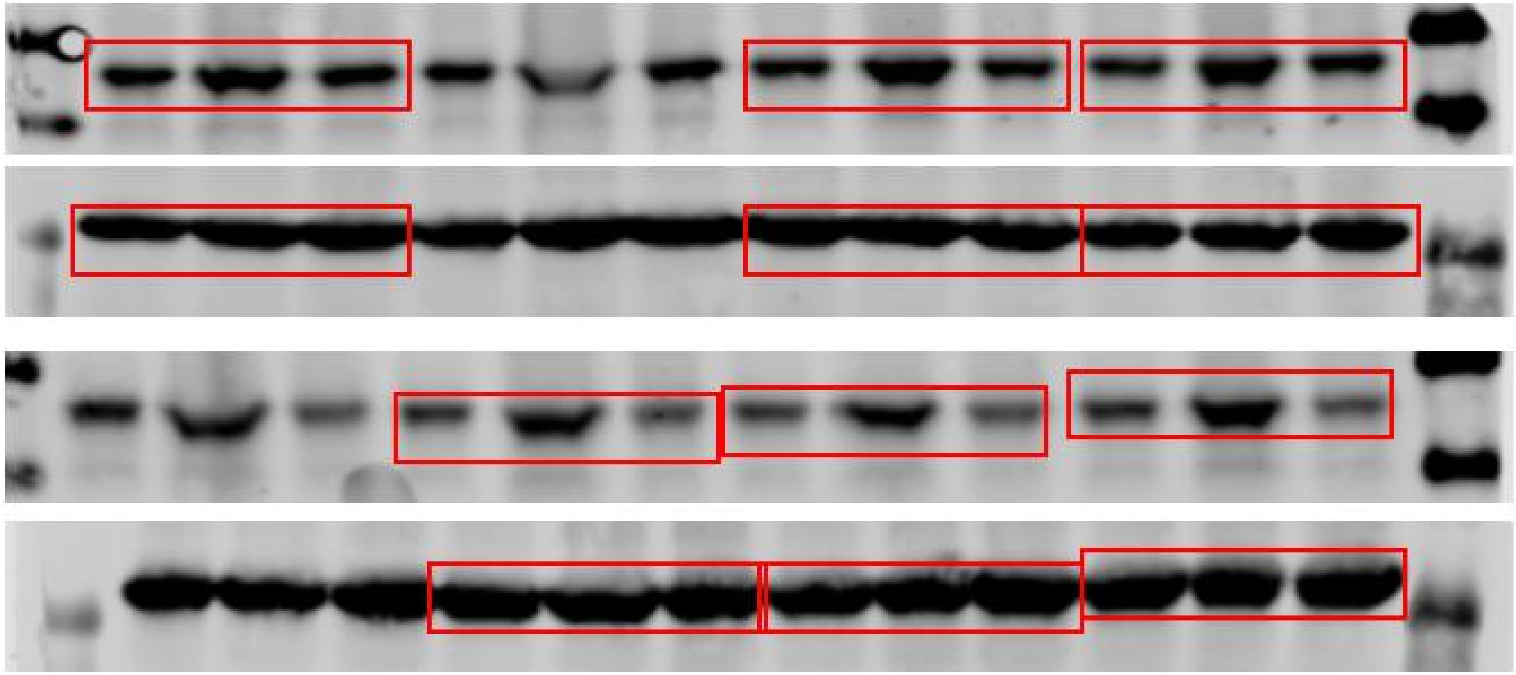

Figure2E-Collagen1

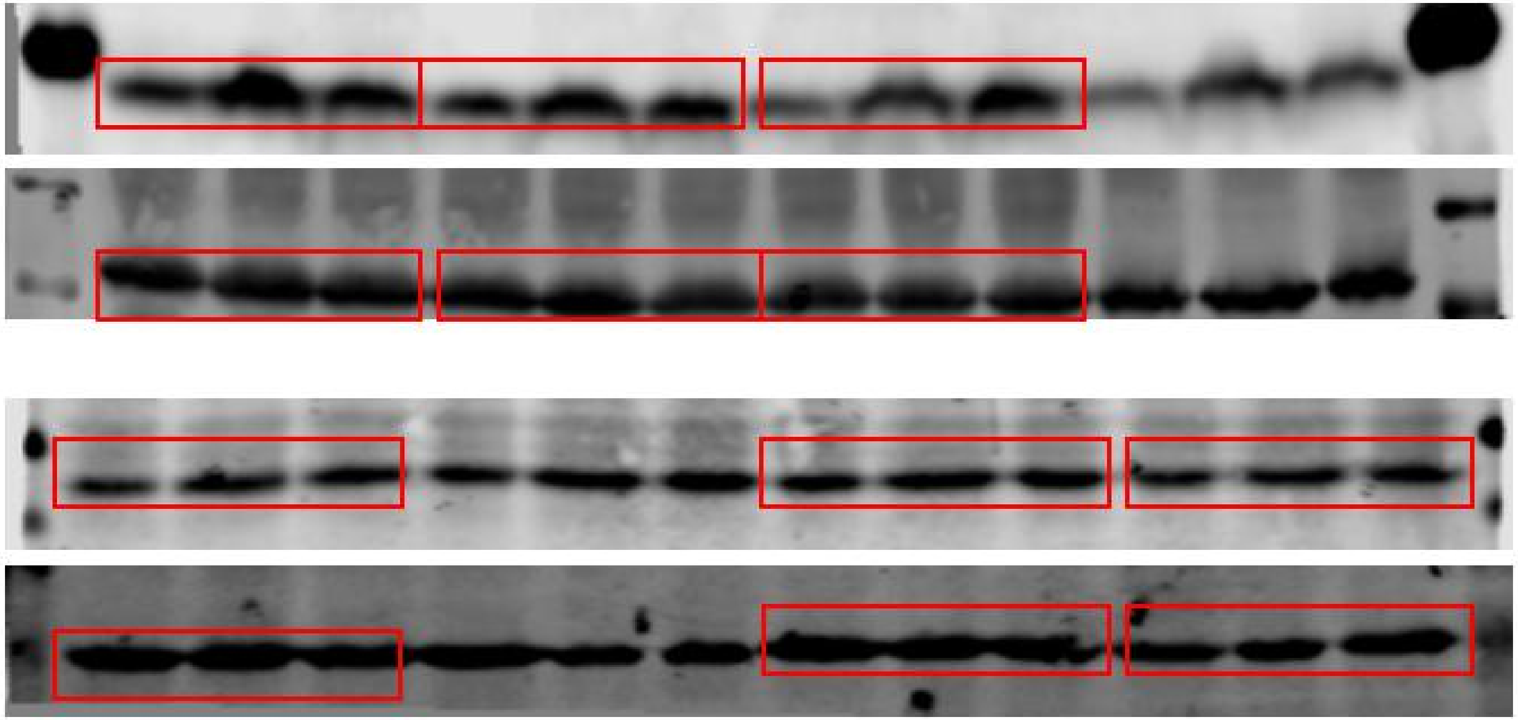

Figure2E-Collagen3

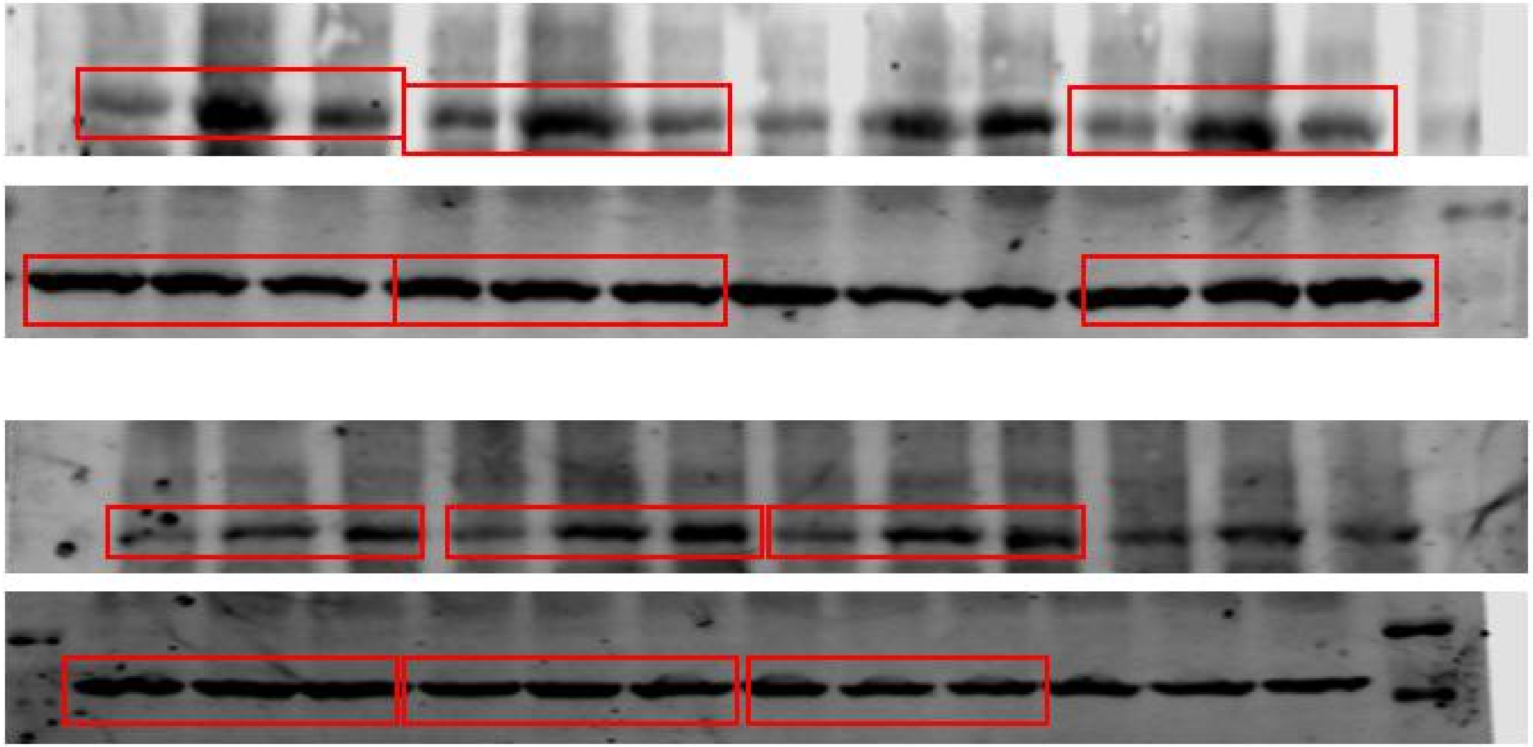

Figure3A

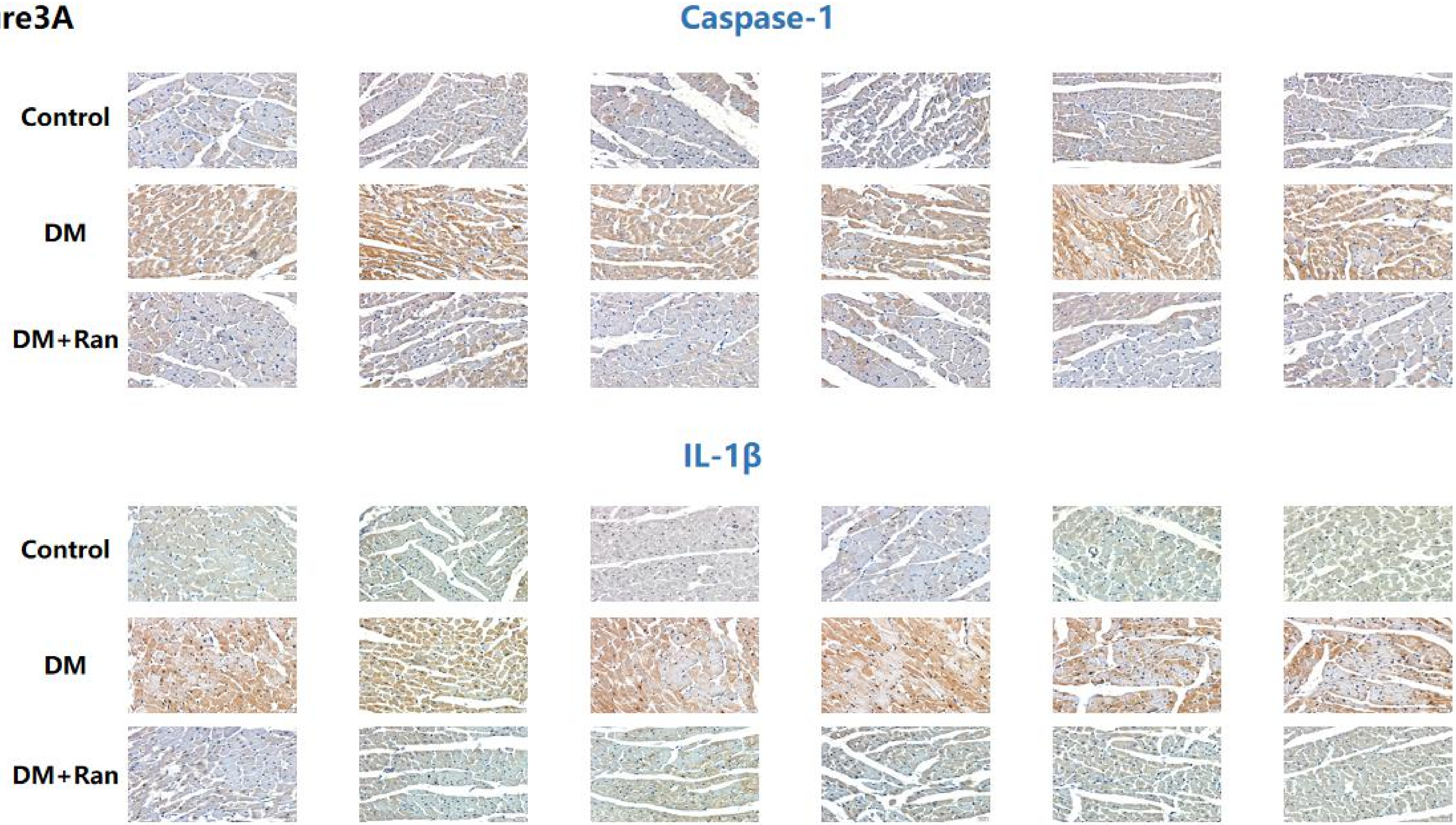

Figure3A

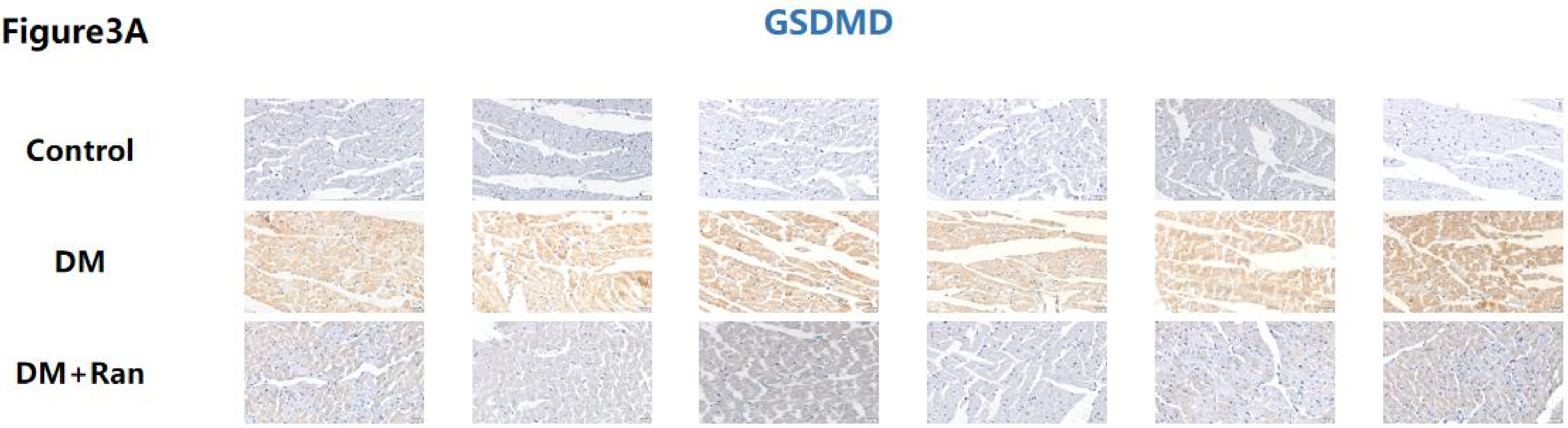

Figure3B-D

	Caspase-1	IL-1β	GSDMD
Control-1	7.18	7.12	3.46
Control-2	7.86	6.79	3.94
Control-3	8.7	9.93	4.85
Control-4	8.2	11.01	2.8
Control-5	9.96	8.14	3.57
Control-6	10.29	8.91	4.36
DM-1	36.38	26.73	27.68
DM-2	39.38	30.5	24.92
DM-3	40.27	32.98	28.18
DM-4	34.85	30.27	22.74
DM-5	38.42	32.54	17.27
DM-6	34.96	28.07	22.98
DM+Ran-1	17.22	14.55	14.36
DM+Ran-2	22.87	14.46	10.6
DM+Ran-3	16.94	13.39	13.95
DM+Ran-4	17.49	15.24	14.81
DM+Ran-5	20.14	12.65	13.39
DM+Ran-6	18.3	15.35	16.81

Figure3E-F

	GAPDH	Caspase-1
Control-1	15.27	25.73
Control-2	15.13	25.75
Control-3	15.29	25.67
Control-4	15.34	25.74
Control-5	15.22	25.69
Control-6	14.27	24.75
DM-1	14.73	23.42
DM-2	14.68	23.39
DM-3	15.86	24.55
DM-4	15.73	24.22
DM-5	15.86	24.65
DM-6	15.47	24.66
DM+Ran-1	15.29	24.76
DM+Ran-2	15.64	24.95
DM+Ran-3	14.51	23.75
DM+Ran-4	15.72	25.31
DM+Ran-5	15.94	25.14
DM+Ran-6	15.36	25.34

	GAPDH	IL-1β
Control-1	14.51	23.86
Control-2	15.13	24.65
Control-3	15.29	24.73
Control-4	15.34	24.81
Control-5	15.22	24.95
Control-6	14.27	23.64
DM-1	14.73	23.19
DM-2	15.49	23.61
DM-3	14.59	22.75
DM-4	15.73	23.82
DM-5	14.27	22.43
DM-6	14.34	22.54
DM+Ran-1	14.29	22.9
DM+Ran-2	15.64	24.09
DM+Ran-3	14.51	22.92
DM+Ran-4	15.72	24.35
DM+Ran-5	14.72	23.23
DM+Ran-6	14.83	23.44

Figure3G-Caspase-1

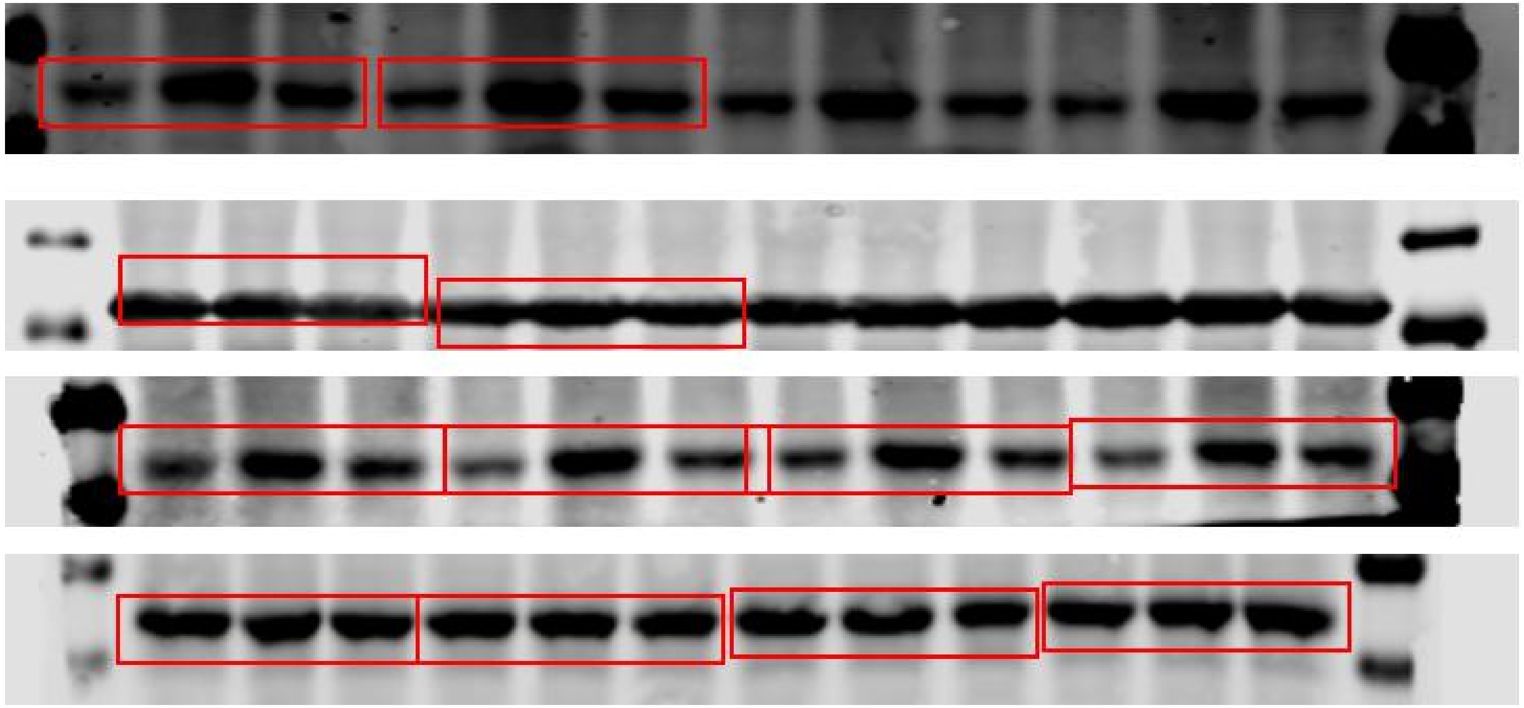

Figure3H-IL-1β

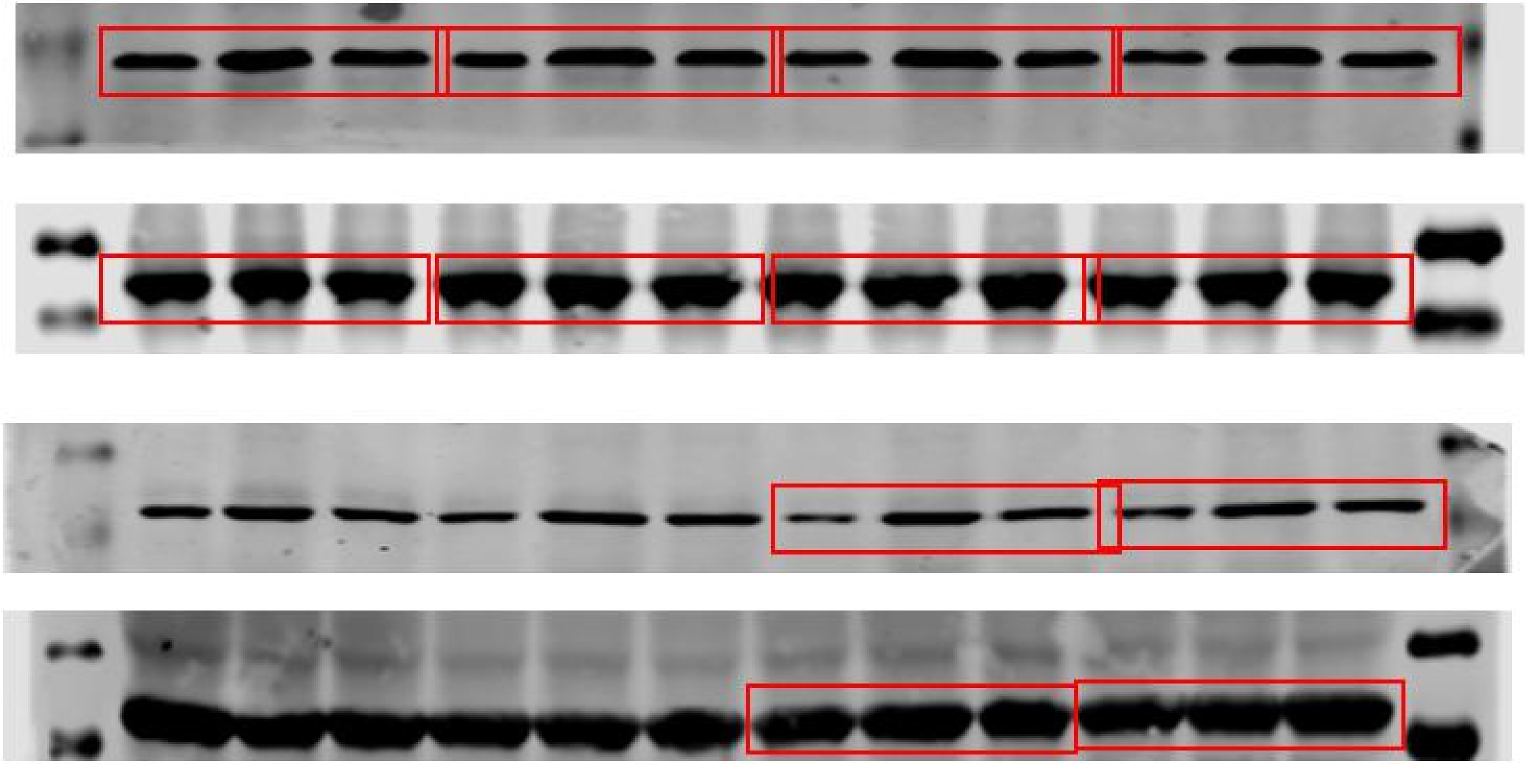

Figure3I-GSDMD

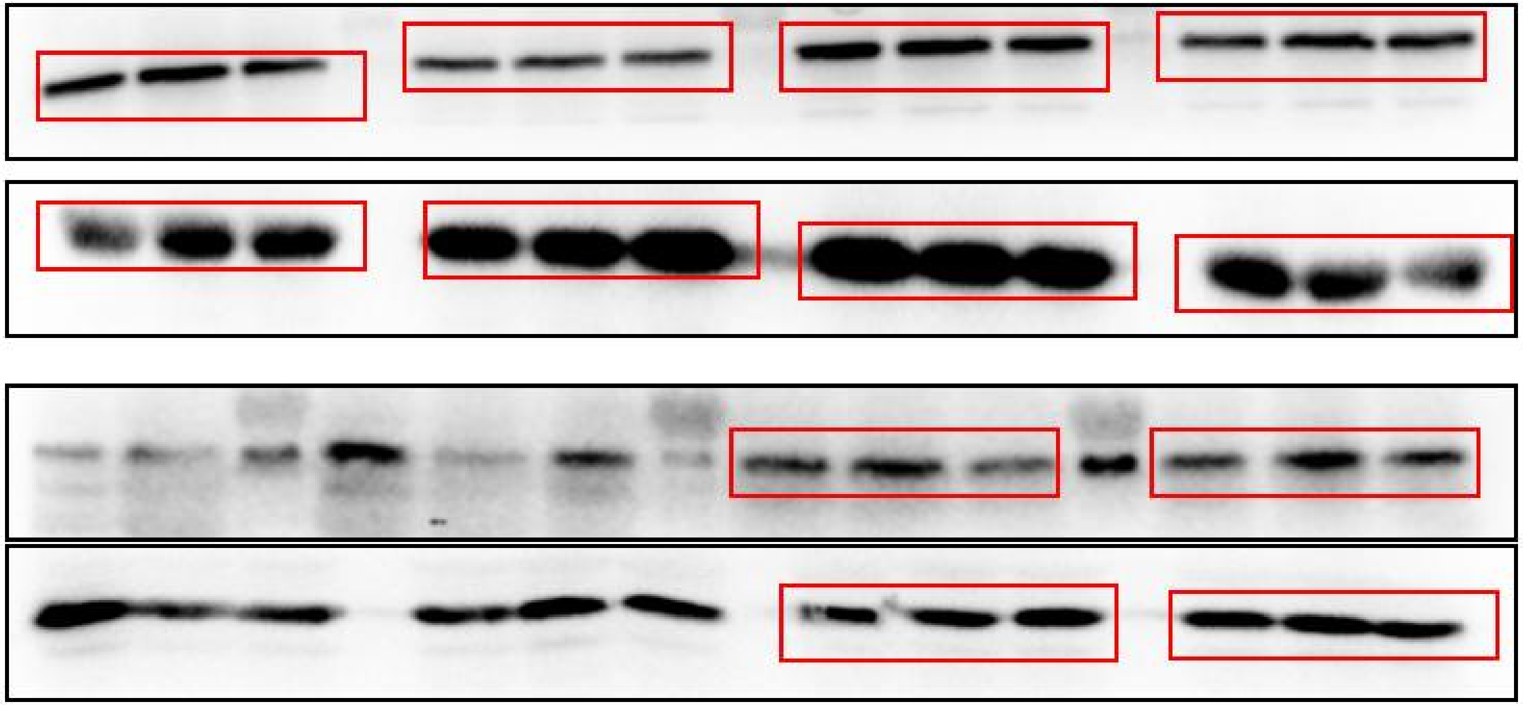

Figure4A

dataset: 1
Target: *CASP1*
length: 1316
MiRNA: *rno-miR-135b*
length: 23

mfe: -21.3 kcal/mol
p-value: undefined

Position: 181

```
target 5'  G     UAAAAA    CCCCG GC        G  3'
             UCAC      AGGA      G  AAGCCA
             AGUG      UCCU      C  UUCGGU
miRNA  3'        UA        UA     UU       AU 5'
```

Caspase-1-WT 5'-- AAAAGGACCCCGGGCAAGCCAGA --3'
Rno-miR-135b 3'-- AGUGUAUCCUUACUUUUCGGUAU --5'
Caspase-1-Mut 5'-- AAAAGGACCCCGGGCGAATGGGA --3'

	miRNA	Background	Firefly	Renilla	R/F
CDS+CDS+3' UTR CASP1	NC1	70	1337104	15952665	11.931
	NC2	73	1442564	16763688	11.621
	NC3	79	1438264	17726661	12.325
	rno-miR-135b 1	80	1219363	7153338	5.866
	rno-miR-135b 2	71	1567536	8919137	5.69
	rno-miR-135b 3	68	1388713	8431935	6.072
CDS+CDS+3' UTR mut CASP1	NC1	81	659318	5109513	7.75
	NC2	86	483149	4014036	8.308
	NC3	92	497855	4063386	8.162
	rno-miR-135b 1	77	692401	5654557	8.167
	rno-miR-135b 2	78	612271	5132015	8.382
	rno-miR-135b 3	73	616980	4948909	8.021

Figure4B-D

	U6	miR-135b
NG+AMO-NC-1	11.21	27.42
NG+AMO-NC-2	11.28	27.45
NG+AMO-NC-3	11.82	27.93
HG+AMO-NC-1	11.9	28.82
HG+AMO-NC-2	11.87	28.92
HG+AMO-NC-3	11.64	28.73
HG+Ran+AMO-NC-1	11.46	27.79
HG+Ran+AMO-NC-2	11.2	27.51
HG+Ran+AMO-NC-3	11.11	27.75
HG+Ran+AMO-135b-1	11.12	29.58
HG+Ran+AMO-135b-2	11.33	29.91
HG+Ran+AMO-135b-3	11.41	29.92

	GAPDH	Caspase-1
NG+AMO-NC-1	17.9	30.03
NG+AMO-NC-2	17.89	30.04
NG+AMO-NC-3	16.21	28.3
HG+AMO-NC-1	17.44	28.08
HG+AMO-NC-2	15.84	26.29
HG+AMO-NC-3	15.86	26.28
HG+Ran+AMO-NC-1	17.33	29.01
HG+Ran+AMO-NC-2	15.82	27.51
HG+Ran+AMO-NC-3	15.76	27.54
HG+Ran+AMO-135b-1	16.22	27.2
HG+Ran+AMO-135b-2	16.23	27.34
HG+Ran+AMO-135b-3	16.26	27.35

	GAPDH	IL-1β
NG+AMO-NC-1	17.9	26.71
NG+AMO-NC-2	17.89	26.66
NG+AMO-NC-3	17.94	26.71
HG+AMO-NC-1	17.44	25.35
HG+AMO-NC-2	15.86	24.02
HG+AMO-NC-3	15.91	24.01
HG+Ran+AMO-NC-1	17.78	26.34
HG+Ran+AMO-NC-2	15.81	24.4
HG+Ran+AMO-NC-3	15.76	24.36
HG+Ran+AMO-135b-1	17.75	25.92
HG+Ran+AMO-135b-2	16.23	24.49
HG+Ran+AMO-135b-3	16.26	24.5

Figure4D-F

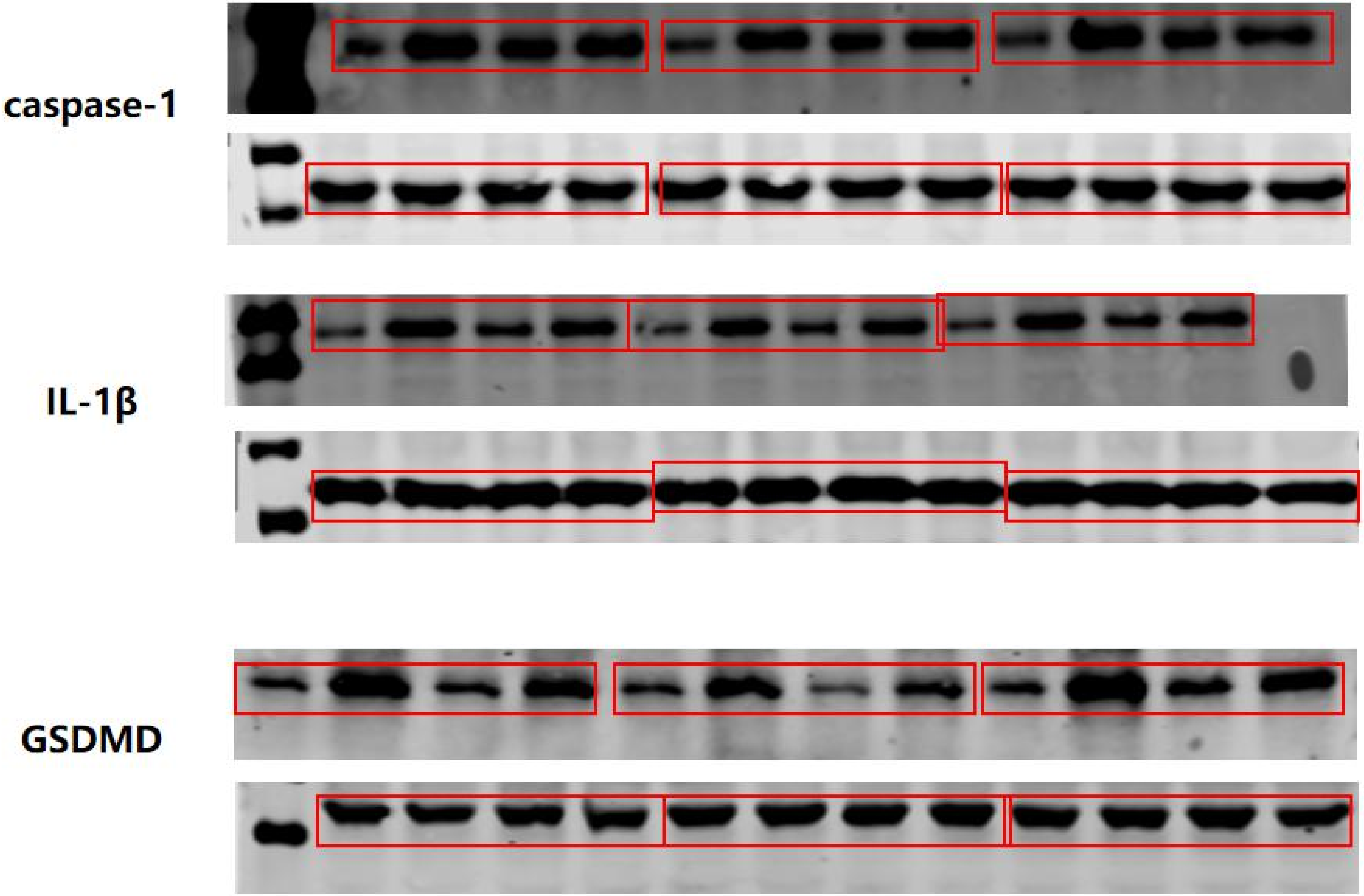

Figure4G

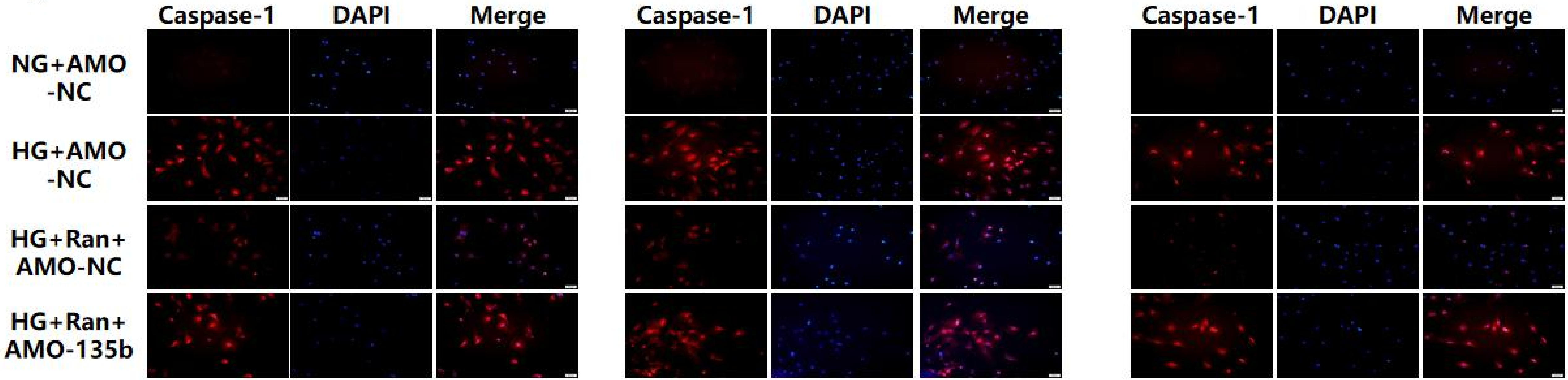

Caspase-1
DAPI
Merge
Caspase-1
DAPI
Merge
Caspase-1
DAPI
Merge
NG+AMO -NC
HG+AMO -NC
HG+Ran+ AMO-NC
HG+Ran+ AMO-135b

Figure4H

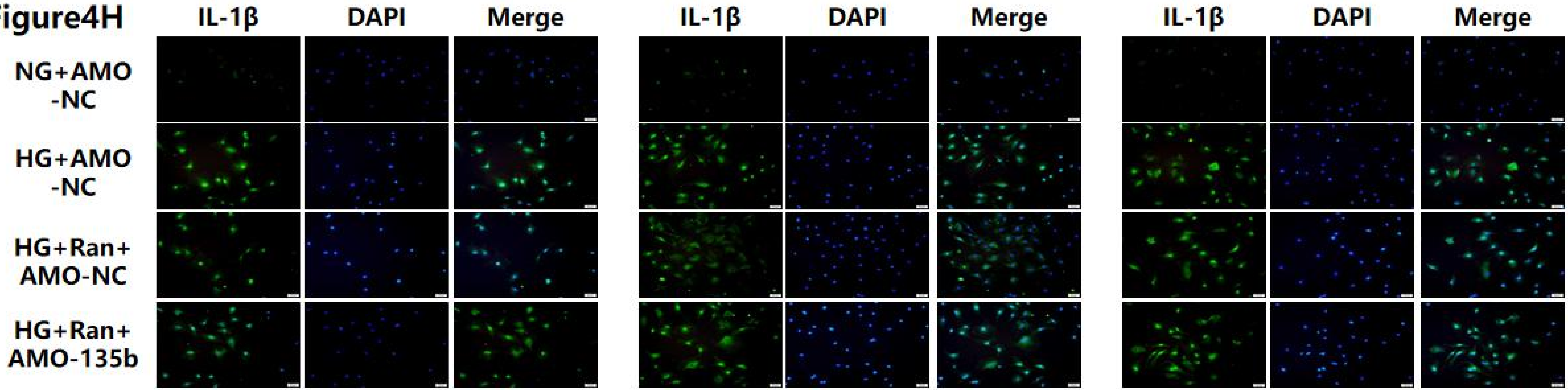

Figure4I

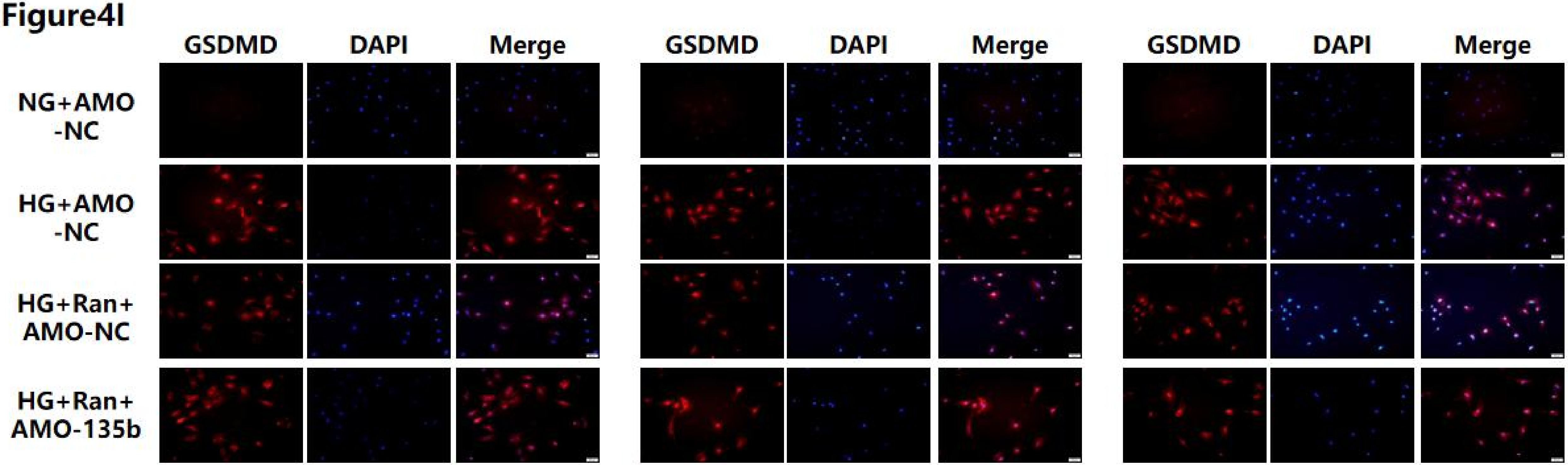

Figure5A

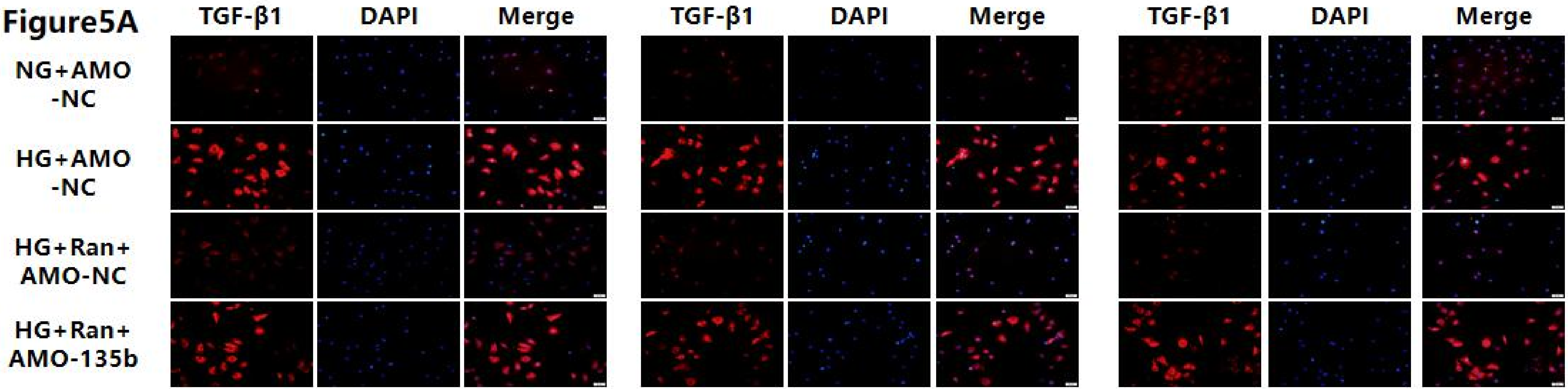

Figure5B

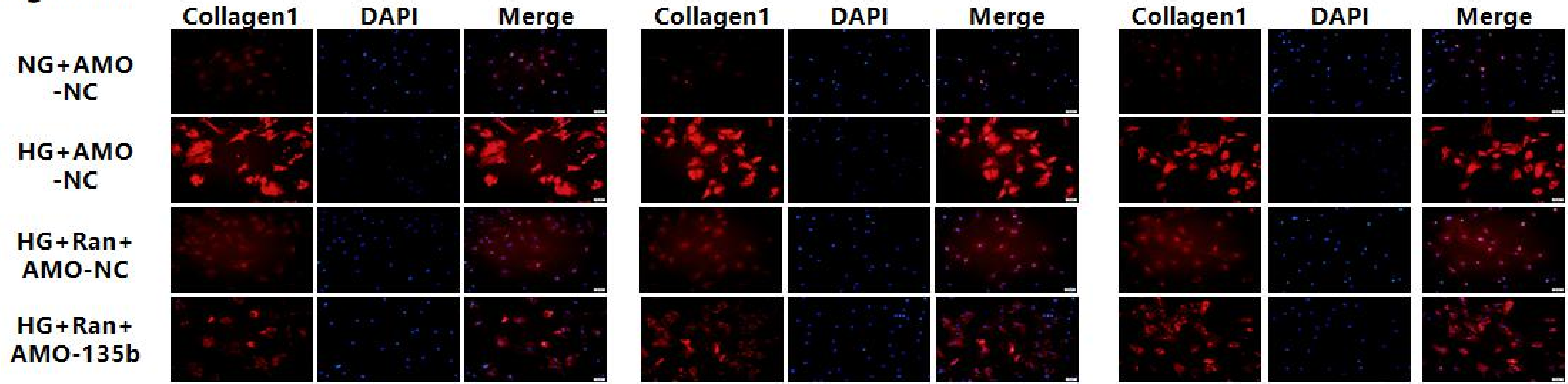

Figure5C

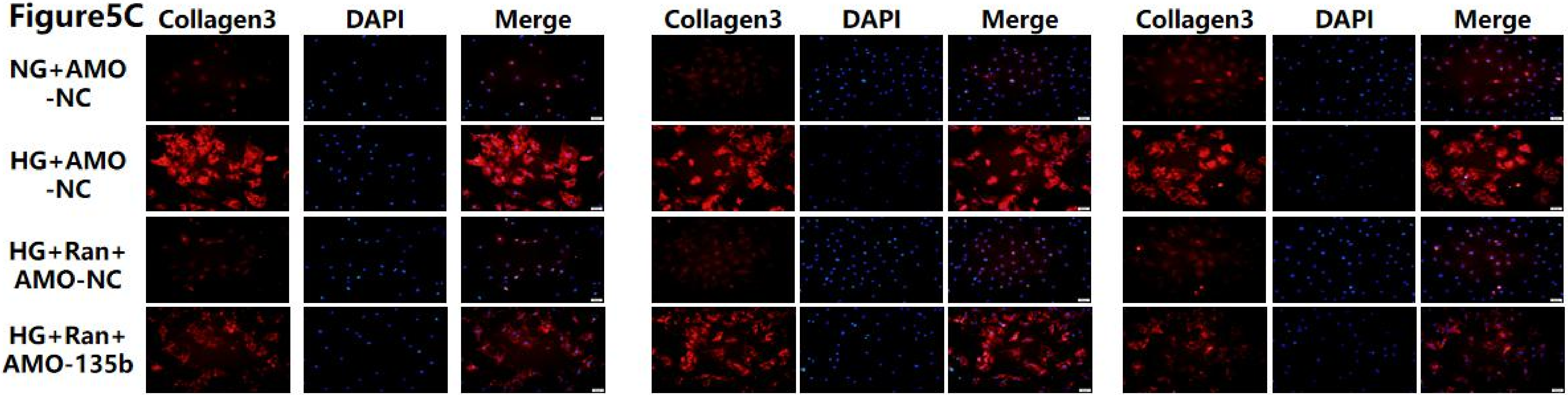

Figure5D-F

	GAPDH	TGF-β1
NG+AMO-NC-1	17.89	29.52
NG+AMO-NC-2	17.94	29.26
NG+AMO-NC-3	16.21	27.56
HG+AMO-NC-1	15.86	26.27
HG+AMO-NC-2	17.44	27.69
HG+AMO-NC-3	15.91	26.22
HG+Ran+AMO-NC-1	18.21	29.08
HG+Ran+AMO-NC-2	17.78	28.73
HG+Ran+AMO-NC-3	15.82	26.55
HG+Ran+AMO-135b-1	17.75	28.34
HG+Ran+AMO-135b-2	16.22	26.88
HG+Ran+AMO-135b-3	16.23	26.75

	GAPDH	Collagen1
NG+AMO-NC-1	17.94	25.92
NG+AMO-NC-2	16.3	24.27
NG+AMO-NC-3	16.25	24.18
HG+AMO-NC-1	17.44	23.51
HG+AMO-NC-2	15.86	22.03
HG+AMO-NC-3	15.91	22.12
HG+Ran+AMO-NC-1	18.21	25.22
HG+Ran+AMO-NC-2	17.78	25.2
HG+Ran+AMO-NC-3	15.76	23.07
HG+Ran+AMO-135b-1	17.75	24.55
HG+Ran+AMO-135b-2	17.79	24.7
HG+Ran+AMO-135b-3	16.26	22.97

	GAPDH	Collagen3
NG+AMO-NC-1	17.9	24.25
NG+AMO-NC-2	17.89	24.34
NG+AMO-NC-3	17.94	24.17
HG+AMO-NC-1	17.44	22.61
HG+AMO-NC-2	15.86	20.91
HG+AMO-NC-3	15.91	20.94
HG+Ran+AMO-NC-1	18.21	23.77
HG+Ran+AMO-NC-2	15.82	21.47
HG+Ran+AMO-NC-3	15.76	21.59
HG+Ran+AMO-135b-1	17.79	23.23
HG+Ran+AMO-135b-2	16.23	21.58
HG+Ran+AMO-135b-3	16.26	21.66

Figure5G-TGF-β1

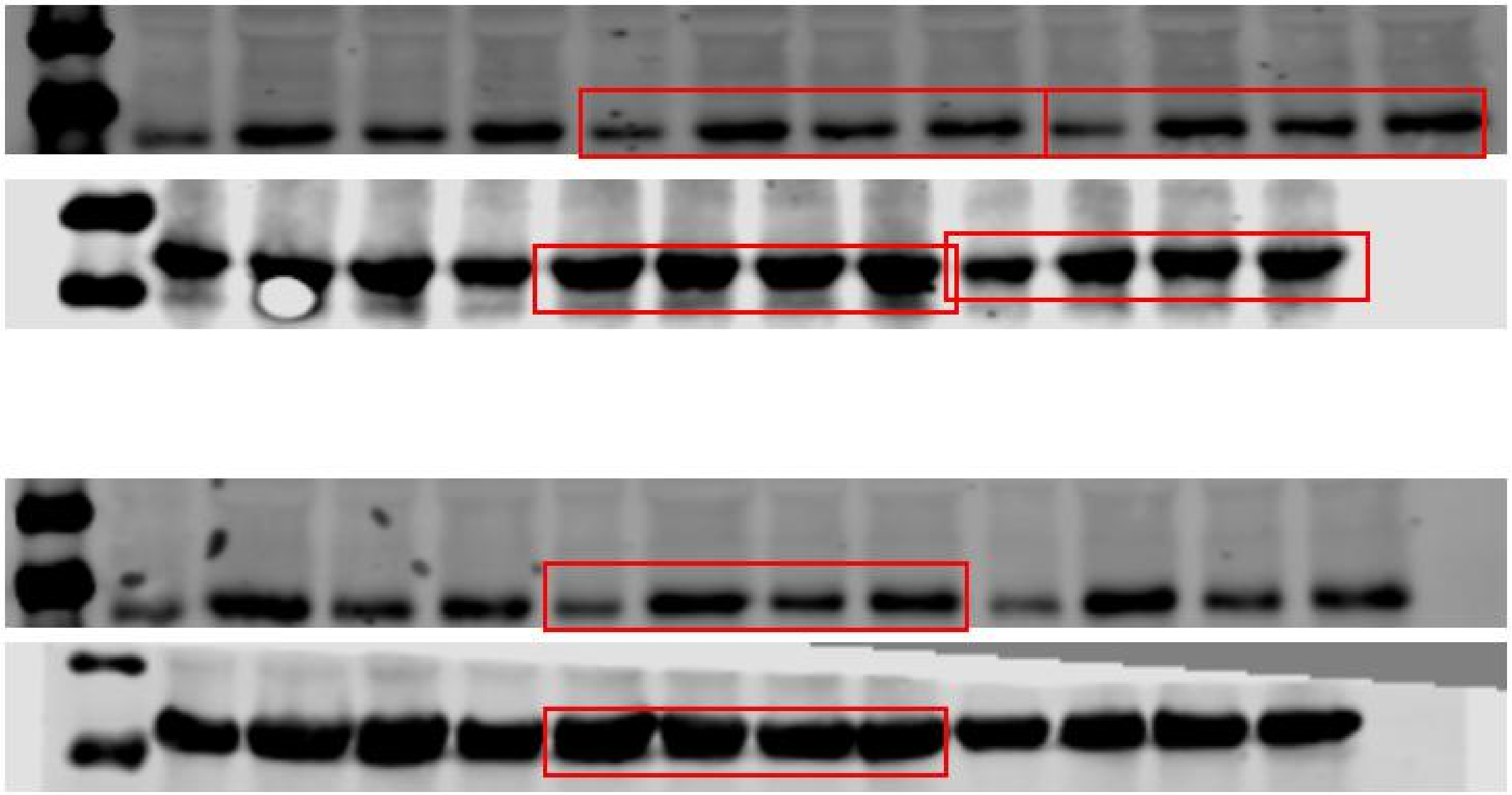

Figure5H-Collagen1

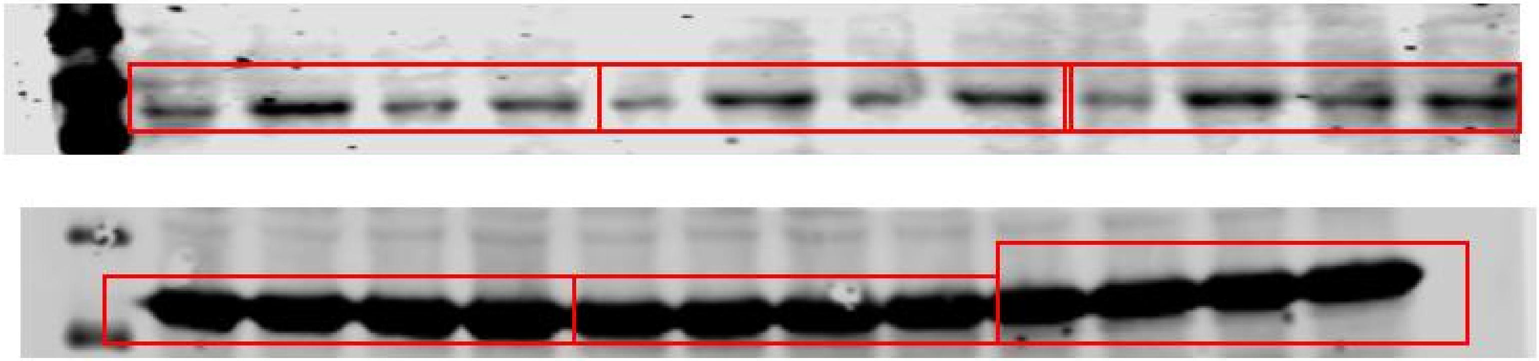

Figure5I-Collagen3

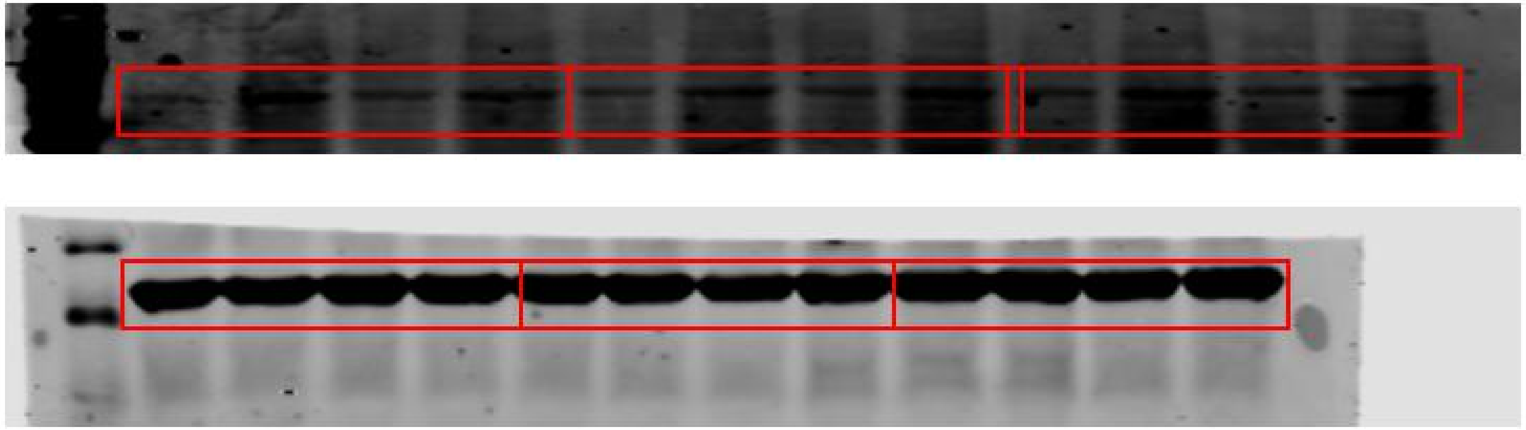

Supplement: Supplementary file 1 [file Image1.pdf]
